# Supplementary material for: Patients’ views about screening for atrial fibrillation (AF): a qualitative study in primary care
Source: BMJ Open. 2020 Mar 18;10(3):e033061. doi: 10.1136/bmjopen-2019-033061 (PMC7150591; doi:10.1136/bmjopen-2019-033061)
Supplement: Author's manuscript [file bmjopen-2019-033061.draft_revisions.pdf]

BMJ Open is committed to open peer review. As part of this commitment we make the peer review history of every article we publish publicly available.

When an article is published we post the peer reviewers' comments and the authors' responses online. We also post the versions of the paper that were used during peer review. These are the versions that the peer review comments apply to.

The versions of the paper that follow are the versions that were submitted during the peer review process. They are not the versions of record or the final published versions. They should not be cited or distributed as the published version of this manuscript.

BMJ Open is an open access journal and the full, final, typeset and author-corrected version of record of the manuscript is available on our site with no access controls, subscription charges or pay-per-view fees (http://bmjopen.bmj.com).

If you have any questions on BMJ Open's open peer review process please email info.bmjopen@bmj.com

BMJ Open

Patients' views about screening for atrial fibrillation (AF): a qualitative study in primary care

Journal:	*BMJ Open*
Manuscript ID	bmjopen-2019-033061
Article Type:	Research
Date Submitted by the Author:	18-Jul-2019
Complete List of Authors:	`Lown, Mark; University of Southampton, Primary Care and Population Sciences<br>Wilcox, Christopher; University of Southampton, Primary Care and Population Sciences<br>hughes, Stephanie; University of Southampton, Primary Care and Population Sciences<br>Santer, Miriam; University of Southampton, Primary Care & Population Sciences<br>Lewith, George; University of Southampton<br>Moore, Michael; University of Southampton Medical School, Primary Care Medical Group<br>Little, Paul; University of Southampton, Primary Care and Population Science;
Keywords:	atrial fibrillation, QUALITATIVE RESEARCH, screening, general practice

SCHOLARONE™
Manuscripts

I, the Submitting Author has the right to grant and does grant on behalf of all authors of the Work (as defined in the below author licence), an exclusive licence and/or a non-exclusive licence for contributions from authors who are: i) UK Crown employees; ii) where BMJ has agreed a CC-BY licence shall apply, and/or iii) in accordance with the terms applicable for US Federal Government officers or employees acting as part of their official duties; on a worldwide, perpetual, irrevocable, royalty-free basis to BMJ Publishing Group Ltd ("BMJ") its licensees and where the relevant Journal is co-owned by BMJ to the co-owners of the Journal, to publish the Work in this journal and any other BMJ products and to exploit all rights, as set out in our licence.

The Submitting Author accepts and understands that any supply made under these terms is made by BMJ to the Submitting Author unless you are acting as an employee on behalf of your employer or a postgraduate student of an affiliated institution which is paying any applicable article publishing charge ("APC") for Open Access articles. Where the Submitting Author wishes to make the Work available on an Open Access basis (and intends to pay the relevant APC), the terms of reuse of such Open Access shall be governed by a Creative Commons licence – details of these licences and which Creative Commons licence will apply to this Work are set out in our licence referred to above.

Other than as permitted in any relevant BMJ Author's Self Archiving Policies, I confirm this Work has not been accepted for publication elsewhere, is not being considered for publication elsewhere and does not duplicate material already published. I confirm all authors consent to publication of this Work and authorise the granting of this licence.

For peer review only

Patients' views about screening for atrial fibrillation (AF): a qualitative study in primary care

Mark Lown, Christopher R Wilcox, Stephanie Hughes, Miriam Santer, George Lewith‡, Michael Moore, Paul Little,

M Lown BEng, PhD, MBBS, MRCGP, GP and Clinical Lecturer, Department of Primary Care and Population Sciences, University of Southampton, Southampton.

CR Wilcox, BSc, MBBCh, Clinical Research Fellow, NIHR Clinical Research Facility, University Hospital Southampton NHS Foundation Trust, UK, Department of Primary Care and Population Sciences, University of Southampton, Southampton

S Hughes, BSc, MSc, Senior Research Assistant, Department of Primary Care and Population Sciences, University of Southampton, Southampton.

M Santer, MBBCHir, MRCGP, PhD, Associate Professor in Primary Care Research, Department of Primary Care and Population Sciences, University of Southampton, Southampton.

G Lewith MA, MD, FRCP, MRCGP, Professor of Primary care Research, Department of Primary Care and Population Sciences, University of Southampton, Southampton.

M Moore, BM, BS, MRCP, MRCGP Professor of Primary care Research, Department of Primary Care and Population Sciences, University of Southampton, Southampton.

P Little, MBBS, BA, MD, DLSHTM, MRCP, FRCGP, FMedSci, Professor of Primary care Research, Department of Primary Care and Population Sciences, University of Southampton, Southampton.

‡Deceased

Address for correspondence:

Mark Lown Department of Primary Care and Population Sciences, Aldermoor Health Centre, Aldermoor Close, University of Southampton, Southampton SO165ST.

Email: m.lown@soton.ac.uk

For peer review only

Abstract

Background

There has been increased interest in screening for Atrial Fibrillation (AF) with commissioned pilot schemes, ongoing large clinical trials and the emergence of inexpensive consumer single-lead ECG devices that can be used to detect AF. This qualitative study aimed to explore patients' views and understanding of AF and AF screening in order to determine acceptability and inform future recommendations.

Method

15 participants were interviewed from primary care who had taken part in an AF screening trial. A semi-structured interview guide was used flexibly, to enable the interviewer to explore any relevant topics raised by the participants. Interviews were recorded, transcribed verbatim, and analysed using inductive thematic analysis.

Results

Participants generally had an incomplete understanding of AF and conflated it with other heart problems or with raised blood pressure. With regards to potential drawbacks from screening, some participants considered anxiety and the cost of implementation, but none acknowledged potential harms associated with screening such as side effects of anti-coagulation treatment or the risk of further investigations. The screening were generally well accepted, and participants were generally in favour of engaging with prolonged screening.

Conclusions

Our study highlights that there may be poor understanding (of both the nature of AF and potential negatives of screening) amongst patients who have been screened for AF. Further work is required to determine if resources including decision aids can address this important knowledge gap, and improve clinical informed consent for AF screening.

For peer review only

Strengths and limitations of this study

- A strength of this study is that all the interviews were conducted by a single researcher, thus ensuring consistency.
- The study had good representation in terms of a typical screening population aged over 65 years, although there were only four male participants and all participants were from a single GP surgery in England.
- All of the participants had taken part in the SAFETY AF screening trial and those not wishing to be screened for atrial fibrillation may have had different views.
- The participants all had a negative screen which may have affected their attitudes.
- The sample size was appropriate and sufficient to achieve saturation.

For peer review only

Introduction

Atrial fibrillation (AF) is a common heart rhythm irregularity, characterised by abnormal beating of the atria, and affects around 10% of people aged over 65 in the UK.[1] AF is associated with an increased risk of stroke which is substantially reduced by anticoagulation.[2] As a significant proportion of AF is paroxysmal and can be asymptomatic, it can often go undiagnosed: across England, it is estimated that 425,000 people are living with undiagnosed AF.[3] There has been much recent debate about screening for AF[4] by expert committees and in UK parliament. Currently, no Randomised Controlled Trial (RCT) evidence exists for systematic AF screening for stroke prevention and no country has yet implemented a systematic screening programme. However, the best available evidence does provide a strong case for screening, and the European Cardiac Society currently recommends opportunistic screening in patients aged > 65 and consideration of systematic screening in patients aged > 75 or in those at higher stroke risk.[5] The UK national screening committee is due to review its decision on AF screening imminently.

NHS England has, however, commissioned pilot AF screening schemes in pharmacies and in flu clinics to 'test the treatments and care models of tomorrow.' [6,7] There are ongoing, large RCTs to investigate the cost-effectiveness of AF screening and the outcomes are eagerly awaited.[8] We are also witnessing a shift in healthcare with increased consumerisation of medical devices and the emergence of relatively inexpensive consumer single-lead ECG devices that can be used for AF detection.[9] Consumer watches are now available with ECG capability and the potential to diagnose AF.[10] Hence, there now exists a great need to ensure patients are well informed before undertaking any form of AF screening and to understand patient attitudes towards screening for AF. There is a sparsity of qualitative data in the literature on patient beliefs and attitudes towards screening for AF and in this article, we report on an interview study that aimed to explore patient views on screening for AF.

Methods

The current qualitative study was nested within the Screening for Atrial Fibrillation using Economical and Accurate TechnologY (SAFETY) study.[11] 418 participants were recruited to the SAFETY trial from three primary care practices in the Wessex area. Individuals aged over 65 years both with and without a coded diagnosis of AF in their medical records were

For peer review only - http://bmjopen.bmj.com/site/about/guidelines.xhtml

invited by their GP to a screening visit in order to test the accuracy of several devices (a blood pressure meter, a single-lead device and two ECG sensing consumer devices) for the detection of AF.

Data collection

For this qualitative study, a convenience sample of 34 of the 418 trial participants not known to have AF from a single GP surgery were invited to take part in an interview. Of these, 15 agreed to participate. We only invited participants who did not have AF as they would likely be more representative of a typical screening population. All the participants had previously been sent information leaflets as part of the trial invitation with detailed information on AF, screening for AF, and treatment options if AF were detected. Interviews were conducted by SH (a senior researcher with considerable qualitative research experience) via telephone, audio-recorded and transcribed verbatim, assigning ID numbers to preserve anonymity. A semi-structured interview guide was used flexibly, to enable the interviewer to explore any relevant topics raised by the participants (Table 1). The interview guide covered topics such as the patient's understanding of AF, views about AF screening (including benefits and drawbacks), opinions about the devices trialled (e.g. device comfort) and opinions about future use of the devices. The interviews were carried out between May 2017 and July 2017 and lasted around 15 minutes.

Data analysis

Interview transcripts were analysed using inductive thematic analysis[12]. Transcripts were read and re-read (ML, SH, CW, MS) to identify codes, which were then organised iteratively into a coding manual. Themes were subsequently developed through further discussions within the team which included a mix of clinicians and academics with varying degrees of experience. We assessed the data for saturation of main themes and searched for disconfirming cases. The sample size was appropriate and sufficient to achieve saturation[13].

Patient and public involvement

Patient and public representatives were involved in the design of the study from the funding application stage and in protocol development. All the study materials were developed with lay input.

For peer review only - http://bmjopen.bmj.com/site/about/guidelines.xhtml

Results

Participant characteristics

Of the 15 participants who took part in the qualitative study, four were male (26.7%) and the average age was 68 years (SD = 2.74). A number of themes emerged relating to their [1] understanding of AF, [2] attitudes to screening (in general and for AF specifically), [3] attitudes to the screening devices tested during the SAFETY study, and [4] their attitudes to undergoing prolonged screening.

Understanding of atrial fibrillation

Participants were asked to describe their understanding of AF. In interpreting responses to this question, it needs to be highlighted that participants had previous received an information sheet, which described AF as 'an irregular heart rhythm that can lead to blood clots forming within the heart which can come loose and cause a stroke.' Despite this, there was considerable confusion about the nature of the condition. Although the majority of participants said they were aware that AF related to a problem with the heart, many seemed unaware that it related to heart rhythm irregularity, and few acknowledged its association with risk of stroke or developing clots.

> *"Something wrong with your heart system…of the atrium and connecting pipes"* [P15]

> "Well, to identify a possible stroke and high blood pressure and possible stroke and heart attack." [P5]

> *"Well if the heart isn't functioning properly and this is a condition that could be picked up…if you have heart problems"* [P65]

Attitudes to screening

When asked for their views regarding screening for atrial fibrillation, many of the participants stated positive opinions about health screening in general, with regards to early detection and saving money for the health service.

> *"I believe in screening as much as possible…anything that helps with picking up conditions, I think, is a good thing"* [P72]

> *"Having more treatments or monitoring available…can't be a bad thing"* [P122]

> *"Saves the health service a hell of a lot of money…any kind of health screening on the NHS system is a very good idea"* [P131]

Many participants raised positive opinions regarding early detection and treatment of AF specifically, and two mentioned prevention of stroke.

> *"I think it's sensible to know if you've got AF, because then it's possible to have some treatment"* [P39]

> In response to the question "What are your thoughts about screening for atrial fibrillation": *"Well I actually think it's a good idea; there's a lot of heart conditions in my own family on my father's side, and so it's no harm to be monitored every so often, along the way"* [P53]

> *"I think it's an excellent idea; better to catch a condition early if you possibly can, especially if people…[are] not aware they have it…if there's medication or other things to stop it…I know there are so many other calls on the Health Service, but I know stroke and so on being so debilitating; if you can avoid any at all, that would be a good thing"* [P125]

Most participants didn't describe any potential downsides to screening for AF. Possible negatives that were raised by participants included anxiety and cost for the health service.

> *"It might make you anxious, but my anxiety on that score would be counteracted by - at least I know and it's now in hand, as opposed to not knowing and then it causing a complication"* [P39]

> *"Perhaps…it might trigger people to be too anxious about their health, perhaps if they were …that sort of person"* [P125]

For peer review only

"I suppose the cost for the NHS but, in the long-term, if you can pick something up early and correct it, it's going to be cheaper than if it's left and then down the road they are going to need more care and intervention" [P72]

"I suspect it's probably desirable…the cost or the complexity might outweigh the benefits; I'm not completely sure on that" [P122]

Participants may have misunderstood the scope of the screening test. One participant felt reassured they had a healthy heart.

"Well I suppose it was a chance to see if my heart was healthy; also it reassured me" [P65]

Attitudes to the screening devices

Some participants stated no particular preference towards any device, and felt that all were comfortable.

"Absolutely no question of them being unpleasant…they were very unintrusive and unobjectionable" [P122]

"They were all comfortable. I could have coped with any of them…if I'd been selected to use a particular type it wouldn't have bothered me" [P72]

Others participants had mixed opinions regarding the user-friendliness of the devices, and stated a preference towards those which were the least uncomfortable, least trouble, and least time-consuming to use (although their opinion as to which device was preferred differed between participants).

"I think probably the one on the finger was most convenient to use. There was another one where you had to put patches on yourself...[which] I would probably have to force myself to do at home…it would just be one of those irritating things to do. There was another one…that every time you used it, you were going to have to turn on the computer, log into a site and it would upload the data onto the site... at a specific and regular time…[which would be] a bit of a faff." [P45]

For peer review only

"They were all fine. I think the most difficult one…where you have all the things put on you…that's the one that takes the time. But all the others seemed very, you know, quick…I think the first one was the simplest, the one with the thumbs" [P76]

"The worst one was the blood pressure cuff which was quite uncomfortable. The one…you wore it round your [rib cage] – you weren't aware you were wearing it and I think that was the best one…The handheld device was quite comfortable but…you were holding it, so whereas wearing the thing around your chest, your hands were free to do other things…and the ECG, obviously, you had to be fully engaged with that and you couldn't do other things" [P125]

Attitudes towards undertaking prolonged screening

Many participants stated that they would be happy to undergo prolonged screening using these (or similar) screening devices if it were recommended by their doctor.

"I just tend to follow advice with that sort of stuff…if it's because there was a need…it would be in my best interest then I would do yes" [P15]

"Even if it was uncomfortable…you would just do what you had to do really…I wouldn't have any hesitation if it was a health matter" [P125]

Others were more reserved about the idea of prolonged screening over a number of weeks or had specific concerns, for instance about the time required and the potential of screening to provoke anxiety or 'take over your life'.

"Well I'm not sure. It depends how many weeks we're talking about…I mean I don't have any other particular issue other than the timing" [P131]

"I'm probably a bit of an anxious person…so I might not be quite at ease…I'm not the sort of person that happily does something and just forgets…it tends to take over your life a bit…so if [it was] for two weeks or so, I might be a bit hesitant" [P130]

Some participants seemed unclear or doubtful about prolonged screening, i.e. they would be happy to test for AF if there was a definitive need identified by their doctor but not just if it was a matter of their age. This suggests that the test, even if they felt the test was tolerable, they may have reservations about doing this in the absence of symptoms.

For peer review only

"I mean if there was a valid reason behind it...if it was just oh you're getting to a certain age and we ought to look at it, I would probably…have a bit of a half-hearted effort at it…but if the doctor…[said that there] might be a problem here and it needs further investigation, then obviously I would take it quite seriously" [P45]

Discussion

In this study, participants seemed to have an incomplete understanding of AF and conflated it with other heart problems or with raised blood pressure. With regards to potential drawbacks from screening, some participants did consider anxiety and the cost of implementation, but none acknowledged potential harms associated with screening such as side effects of anti-coagulation treatment, the risk of further investigations, or the accuracy of the diagnostic test and potential for false positive or false negative results. The screening devices in the SAFETY trial were generally well accepted, however some participants preferred unobtrusive and more user-friendly devices. Participants were generally in favour of engaging with prolonged screening (for several weeks) although some said they would only do so if advised by a health care professional or if they had a 'health problem', and had concerns about time requirement and the potential for the testing to provoke anxiety.

Despite the fact that participants had received printed information sheets as part of the SAFETY trial (which included information on AF [including paroxysmal AF], its diagnosis, and anticoagulation treatment), they still appeared to have limited understanding of AF – a finding which is consistent with recent literature: previous studies have identified that many patients with AF were not aware of the name of the condition[14] or that it led to an increased risk of stroke.[15] Other work has found that patients were uncertain what AF was before and after out-patient cardiology clinic appointments.[16] They also had difficulty understanding why they were treated with anti-coagulation, and why treatment was recommended lifelong.[16] Older patients, in particular, may have a poor understanding of AF, which in turn may have a negative impact on their life.[17] Furthermore, a lack of knowledge may be a key barrier to accepting anti-coagulation treatment and future adherence - which is imperative for stroke risk reduction.[18]

For peer review only

Some participants did mention the possibility that screening might provoke anxiety, and although there is a lack of evidence of this for AF screening specifically, data does suggest that screening can induce anxiety in breast and cervical screening programs.[19-20] The literature also suggests that there is potential for psychological harm from being labelled with an unexpected diagnosis through screening for other conditions.[21] However, when directly asked about potential downsides of screening in this study, participants did not mention the potential risk of harm from treatment, or the risk of further investigations when deciding to participate, suggesting that this was not a major concern for them. This is consistent with other studies showing that many patients are unable to identify potential harms from screening tests, and of those that did, they were mostly related to the test itself, not to further testing or treatment.[22] In contrast, patients could name benefits and tended to overestimate them.[22]

Importantly, when asked for their opinions on any potential drawbacks about screening in general, participants did not mention the accuracy of the diagnostic test or the possibility for false positive and/or negative diagnoses, or discuss the potential for over-diagnosis. Again, there is no AF-specific data in the literature and a general lack of understanding of AF and treatment options could account for the lack of concerns regarding diagnostic accuracy. Other work has found many AF patients were unaware that AF could be asymptomatic and therefore they may not be aware of paroxysmal episodes which could remain undiagnosed.[23] Patients do have worry about potential false positive results for other screening tests such as lung cancer tests.[24] There is also concern that patients may not understand the concept of over-diagnosis[19]. However, evidence suggests that older patients may be suspicious or resistant to the concept of over-detection of other conditions.[19,25] Interestingly, there is also evidence that older adults perceived overuse to have occurred when interventions were used in the absence of symptoms (excluding cancer screening), did not improve symptoms, or against their preferences.[26]

The screening devices were generally well accepted by the participants and found to be unobtrusive, but some participants did express concerns about comfort, user-friendliness and time taken to use the device. AF screening devices have been found to be well-accepted by participants in previous large-scale trials.[27,28] However, many participants stated they would be happy to undergo prolonged screening only if recommended by their doctor and

For peer review only

some participants had specific concerns with respect to the time taken and the potential to provoke anxiety.

Practice and policy implications

There is a need to provide clear and concise information about AF, and to check patient understanding, before proceeding with any form of screening. Checklists could be used to ensure key points have been discussed and considered, and patients may require time to weigh up the risks and benefits before deciding to proceed with screening. Decision aids have been implemented for AF treatment but have usually been designed to support clinician decisions and do not explicitly engage patients.[29] Decision aids could potentially improve patient knowledge prior to screening and have been used to improve knowledge for other screening programs.[30-33] Good patient knowledge is required in order to ensure shared decision-making about screening can take place. Health care professionals could actively ask about any potential anxiety participants may have prior to screening and be prepared to discuss these.

Consent for screening rests on having sufficient information about the procedure and its potential downsides. Adequate information prior to screening should include the risk of bleeding with anticoagulation treatment if AF were detected. A discussion of available treatment options and lifestyle modifications before undergoing testing might also ensure the potential for future compliance if AF was indeed diagnosed. Patients should also be informed about the specificity of the screening test, and reminded that screening will not provide information on general heart function or cardiovascular risk in order to avoid false reassurance.

Patients should be well- informed regarding the test accuracy and the potential for false negatives which could increase compliance if prolonged or repeated screening were offered. An understanding of the paroxysmal or asymptomatic nature of AF in patients with intermittent AF may enhance treatment adherence. If patients have concerns over the time and effort required for prolonged screening, perhaps discussion of individual risks and benefits before proceeding could aid decision-making. As participants expressed opinions on the user-friendliness and comfort of devices, these issues should be considered whenever new devices are considered for AF screening or detection.

For peer review only

The emergence of consumer ECG devices and wearable technology such as watches that can detect pulse irregularity and ECG sensing capability[4], will inevitably lead to an increase in asymptomatic patients presenting with suspected irregular pulses or subclinical AF, and we should ensure patients are well informed before referring for further tests (or to disregard this functionality until it has been further assessed). Ideally, patients with clinically detected pulse irregularities should also be fully informed before having further tests. Furthermore, given the lack of RCT evidence for screening and the heterogeneity in stroke risk estimation data,[34] clinicians may be required to have discussions around uncertainty and over-diagnosis, particularly in asymptomatic patients who have effectively self-screened themselves for AF.

Conclusions

Our study highlights that there may be poor understanding (of both the nature of AF and potential negatives of screening) amongst patients who have been screened for AF. Further work is required to determine if resources including decision aids can address this important knowledge gap, and improve clinical informed consent for AF screening.

Word count 3247

Acknowledgements: The authors would like to thank all the participants who took part in this study: without them this study would not have been possible.

Contributions: ML, GL, MM, PL designed the study. SH conducted the interviews. ML & CW coded and analysed the interview data. All authors contributed to the interpretation of the data. ML drafted the manuscript and all authors contributed to the review and editing of the manuscript.

Funding: This study/project is funded by the National Institute for Health Research (NIHR) School for Primary Care Research (project reference 318). The views expressed are those of the authors and not necessarily those of the NIHR or the Department of Health and Social Care.

For peer review only

Competing Interests: None declared.

Ethics approval: The study complies with the declaration of Helsinki, and the protocol was approved by the London - City & East Research Ethics Committee in June 2016 (ref 16/LO/1173). Informed consent was obtained from all participants (trial registration ISRCTN: 17495003).

References

1. Cowan C, Healicon R, Robson I, et al. The use of anticoagulants in the management of atrial fibrillation among general practices in England. *Heart* 2013; **99(16)**: 1166-72.
2. Hart RG, Pearce LA, Aguilar MI. Meta-analysis: antithrombotic therapy to prevent stroke in patients who have nonvalvular atrial fibrillation. *Ann Intern Med.* 2007; **146(12)**:857-67.
3. Reiffel JA, Atrial fibrillation and stroke: epidemiology. *Am J Med.* 2014; **127(4)**:e15-6.
4. Lown M, Moran P. Should we screen for atrial fibrillation? *BMJ.* 2019 Feb 13;364:l43.
5. Mairesse GH, Moran P, Van Gelder IC, Elsner C, Rosenqvist M, Mant J, Banerjee A, Gorenek B, Brachmann J, Varma N, Glotz de Lima G, Kalman J, Claes N, Lobban T, Lane D, Lip GYH, Boriani G; ESC Scientific Document Group. Screening for atrial fibrillation: a European Heart Rhythm Association (EHRA) consensus document endorsed by the Heart Rhythm Society (HRS), Asia Pacific Heart Rhythm Society (APHRS), and Sociedad Latinoamericana de Estimulación Cardíaca y Electrofisiología (SOLAECE). *Europace.* 2017; **19(10)**:1589-1623.
6. Care City launches life-saving atrial fibrillation pathway. https://nhsaccelerator.com/care-city-launches-life-saving-atrial-fibrillation-pathway/. Accessed 01/03/2019.
7. Atrial Fibrillation Check in Influenza Vaccination Programme. http://www.londonscn.nhs.uk/wp-content/uploads/2017/07/heywood-middleton-and-rochdale-ccg.pdf Accessed 01/03/2019.
8. Nuffield Department of Primary Care Health Sciences. Upcoming trial involving 120 000 patients to investigate screening for atrial fibrillation. https://www.phc.ox. ac.uk/news/new-research-to-investigate-whether-screening-can-prevent-one-in-ten-strokes.Accessed 01/03.2019.
9. Gropler MRF, Dalal AS, Van Hare GF, Silva JNA. Can smartphone wireless ECGs be used to accurately assess ECG intervals in pediatrics? A comparison of mobile health monitoring to standard 12-lead ECG. *PLoS One*. 2018;**13(9)**:e0204403.
10. Apple Watch Series 4: Proactive Health Monitor. https://www.apple.com/apple-watch-series-4/health/ accessed 01/04/2019.
11. Lown M, Yue A, Lewith G, Little P, Moore M. Screening for Atrial Fibrillation using Economical and accurate TechnologY (SAFETY)-a pilot study. *BMJ Open*. 2017;**7(1)**:e013535.
12. Braun V, Clarke V. Using Thematic Analysis in Psychology. *Qualitative Research in Psychology* 2006. **3(2)**:77-101
13. Guest G, Bruce A, Johnson L. How many interviews are enough? An experiment with data saturation and variability. *Field Methods* 2006; **18(1)**: 59-82.
14. Lip GY, Kamath S, Jafri M, Mohammed A, Bareford D. Ethnic differences in patient perceptions of atrial fibrillation and anticoagulation therapy: the West Birmingham Atrial Fibrillation Project. *Stroke.* 2002; **33(1)**:238-42
15. McCabe PJ, Schad S, Hampton A, Holland DE. Knowledge and self-management behaviors of patients with recently detected atrial fibrillation. *Heart Lung.* 2008; **37(2)**:79-90.
16. Thrysoee L, Strömberg A, Brandes A, Hendriks JM. Management of newly diagnosed atrial fibrillation in an outpatient clinic setting-patient's perspectives and experiences. *J Clin Nurs.* 2018; **27(3-4)**:601-611.

For peer review only

17. Lindberg T, Sanmartin Berglund J, Elmståhl S, Bohman DM. Older individuals' need for knowledge and follow-up about their chronic atrial fibrillation, lifelong medical treatment and medical controls. *Scand J Caring Sci.* 2017; **31(4)**:1022-1030.
18. Salmasi S, Kwan L, MacGillivray J, Bansback N, De Vera MA, Barry AR, Harrison MJ, Andrade J, Lynd LD, Loewen P. Assessment of atrial fibrillation patients' education needs from patient and clinician perspectives: A qualitative descriptive study. *Thromb Res.* 2019;**173**:109-116.
19. Mathioudakis AG, Salakari M, Pylkkanen L, Saz-Parkinson Z, Bramesfeld A, Deandrea S, Lerda D, Neamtiu L, Pardo-Hernandez H, Solà I, Alonso-Coello P. Systematic review on women's values and preferences concerning breast cancer screening and diagnostic services. *Psychooncology*. 2019 Feb 27.
20. Patel H, Moss EL, Sherman SM. HPV primary cervical screening in England: Women's awareness and attitudes. *Psychooncology*. 2018; **27(6)**:1559-1564.
21. Cotter AR, Vuong K, Mustelin L, Yang Y, Rakhmankulova M, Barclay CJ, Harris RP. Do psychological harms result from being labelled with an unexpected diagnosis of abdominal aortic aneurysm or prostate cancer through screening? A systematic review. *BMJ Open*. 2017; **7(12)**:e017565
22. Sutkowi-Hemstreet A, Vu M, Harris R, Brewer NT, Dolor RJ, Sheridan SL. Adult Patients' Perspectives on the Benefits and Harms of Overused Screening Tests: a Qualitative Study. *J Gen Intern Med.* 2015; **30(11):**1618-26.
23. Salmasi S, De Vera MA, Barry A, Bansback N, Harrison M, Lynd LD, Loewen PS. Assessment of Condition and Medication Knowledge Gaps Among Atrial Fibrillation Patients: A Systematic Review and Meta-analysis. *Ann Pharmacother*. 2019 Mar 2.
24. Carter-Harris L, Brandzel S, Wernli KJ, Roth JA, Buist DSM. A qualitative study exploring why individuals opt out of lung cancer screening. *Fam Pract.* 2017;**34(2)**:239-244
25. Pappadis MR, Volk RJ, Krishnan S, Weller SC, Jaramillo E, Hoover DS, Giordano SH, Tan A, Sheffield KM, Housten AJ, Goodwin JS. Perceptions of overdetection of breast cancer among women 70 years of age and older in the USA: a mixed-methods analysis. *BMJ Open*. 2018**; 8(6)**:e022138.
26. Green AR, Tung M, Segal JB. Older Adults' Perceptions of the Causes and Consequences of Healthcare Overuse: A Qualitative Study. *J Gen Intern Med*. 2018;**33(6)**:892-897.
27. Lowres N, Krass I, Neubeck L, Redfern J, McLachlan AJ, Bennett AA, Freedman SB. Atrial fibrillation screening in pharmacies using an iPhone ECG: a qualitative review of implementation. *Int J Clin Pharm.* 2015;**37(6)**:1111-20.
28. Svennberg E, Engdahl J, Al-Khalili F, Friberg L, Frykman V, Rosenqvist M. Mass Screening for Untreated Atrial Fibrillation: The STROKESTOP Study. *Circulation*. 2015;**131(25)**:2176-84.
29. Probst MA, Noseworthy PA, Brito JP, Hess EP. Shared Decision-Making as the Future of Emergency Cardiology. *Can J Cardiol*. 2018;**34(2)**:117-124.
30. Perestelo-Perez L, Rivero-Santana A, Torres-Castaño A, Ramos-Garcia V, Alvarez-Perez Y, Gonzalez-Hernandez N, Buron A, Pignone M, Serrano-Aguilar P. Effectiveness of a decision aid for promoting colorectal cancer screening in Spain: a randomized trial. *BMC Med Inform Decis Mak.* 2019;**19(1)**:8.
31. Smith SK, Cai A, Wong M, Sousa MS, Peate M, Welsh A, Meiser B, Kaur R, Halliday J, Lewis S, Trevena L, Yanes T, Barlow-Stewart K, Barclay M. Improving women's knowledge about prenatal screening in the era of non-invasive prenatal testing for Down syndrome - development and acceptability of a low literacy decision aid. *BMC Pregnancy Childbirth*. 2018;**18(1)**:499.
32. Wang Y, Anazodo A, Logan S. Systematic review of fertility preservation patient decision aids for cancer patients. *Psychooncology*. 2019 Mar;**28(3)**:459-467.
33. Ivlev I, Jerabkova S, Mishra M, Cook LA, Eden KB. Prostate Cancer Screening Patient Decision Aids: A Systematic Review and Meta-analysis. *Am J Prev Med.* 2018;**55(6)**:896-907
34. Quinn GR, Severdija ON, Chang Y, Singer DE Wide variation in reported rates of stroke across cohorts of patients with atrial fibrillation. *Circulation* 2017*;***135***:*208-19.

For peer review only

**Interview Schedule**
Can you describe your understanding of atrial fibrillation?
What are your views about screening for atrial fibrillation?<br>- What do you think the positives of screening are?<br>- What do you think the negatives of screening are?
What did you think about the study information given to you?
Did you have any reservations about taking part, and if so, can you tell me a bit about them?
Do you have any regrets about taking part? If so, can you tell me a bit about them?
Could you tell me a bit about the devices you tried as part of the SAFETY trial?
How comfortable did you find the devices?
How would you feel about wearing or using the devices for a few weeks for screening?
Is there anything else you'd like to share about your participation in the trial, or your thoughts about AF

Table 1 – Interview Schedule

For peer review only

Standards for Reporting Qualitative Research (SRQR)*

http://www.equator-network.org/reporting-guidelines/srqr/

Title and abstract

	Page/line no(s).
**Title** - Concise description of the nature and topic of the study Identifying the study as qualitative or indicating the approach (e.g., ethnography, grounded theory) or data collection methods (e.g., interview, focus group) is recommended	1
**Abstract** - Summary of key elements of the study using the abstract format of the intended publication; typically includes background, purpose, methods, results, and conclusions	2

Introduction

**Problem formulation** - Description and significance of the problem/phenomenon studied; review of relevant theory and empirical work; problem statement	4
**Purpose or research question** - Purpose of the study and specific objectives or questions	4

Methods

**Qualitative approach and research paradigm** - Qualitative approach (e.g., ethnography, grounded theory, case study, phenomenology, narrative research) and guiding theory if appropriate; identifying the research paradigm (e.g., postpositivist, constructivist/ interpretivist) is also recommended; rationale**	4-5
**Researcher characteristics and reflexivity** - Researchers' characteristics that may influence the research, including personal attributes, qualifications/experience, relationship with participants, assumptions, and/or presuppositions; potential or actual interaction between researchers' characteristics and the research questions, approach, methods, results, and/or transferability	5
**Context** - Setting/site and salient contextual factors; rationale**	5
**Sampling strategy** - How and why research participants, documents, or events were selected; criteria for deciding when no further sampling was necessary (e.g., sampling saturation); rationale**	5
**Ethical issues pertaining to human subjects** - Documentation of approval by an appropriate ethics review board and participant consent, or explanation for lack thereof; other confidentiality and data security issues	13
**Data collection methods** - Types of data collected; details of data collection procedures including (as appropriate) start and stop dates of data collection and analysis, iterative process, triangulation of sources/methods, and modification of procedures in response to evolving study findings; rationale**	5

For peer review only - http://bmjopen.bmj.com/site/about/guidelines.xhtml

**Data collection instruments and technologies** - Description of instruments (e.g., interview guides, questionnaires) and devices (e.g., audio recorders) used for data collection; if/how the instrument(s) changed over the course of the study	5
**Units of study** - Number and relevant characteristics of participants, documents, or events included in the study; level of participation (could be reported in results)	5
**Data processing** - Methods for processing data prior to and during analysis, including transcription, data entry, data management and security, verification of data integrity, data coding, and anonymization/de-identification of excerpts	5
**Data analysis** - Process by which inferences, themes, etc., were identified and developed, including the researchers involved in data analysis; usually references a specific paradigm or approach; rationale**	5
**Techniques to enhance trustworthiness** - Techniques to enhance trustworthiness and credibility of data analysis (e.g., member checking, audit trail, triangulation); rationale**	5

Results/findings

**Synthesis and interpretation** - Main findings (e.g., interpretations, inferences, and themes); might include development of a theory or model, or integration with prior research or theory	6-9
**Links to empirical data** - Evidence (e.g., quotes, field notes, text excerpts, photographs) to substantiate analytic findings	6-9

Discussion

**Integration with prior work, implications, transferability, and contribution(s) to the field -** Short summary of main findings; explanation of how findings and conclusions connect to, support, elaborate on, or challenge conclusions of earlier scholarship; discussion of scope of application/generalizability; identification of unique contribution(s) to scholarship in a discipline or field	10-13
**Limitations** - Trustworthiness and limitations of findings	3

Other

**Conflicts of interest** - Potential sources of influence or perceived influence on study conduct and conclusions; how these were managed	13
**Funding** - Sources of funding and other support; role of funders in data collection, interpretation, and reporting	13

*The authors created the SRQR by searching the literature to identify guidelines, reporting standards, and critical appraisal criteria for qualitative research; reviewing the reference lists of retrieved sources; and contacting experts to gain feedback. The SRQR aims to improve the transparency of all aspects of qualitative research by providing clear standards for reporting qualitative research.

For peer review only - http://bmjopen.bmj.com/site/about/guidelines.xhtml

**The rationale should briefly discuss the justification for choosing that theory, approach, method, or technique rather than other options available, the assumptions and limitations implicit in those choices, and how those choices influence study conclusions and transferability. As appropriate, the rationale for several items might be discussed together.

Reference:

O'Brien BC, Harris IB, Beckman TJ, Reed DA, Cook DA. **Standards for reporting qualitative research: a synthesis of recommendations.** *Academic Medicine*, Vol. 89, No. 9 / Sept 2014
DOI: 10.1097/ACM.0000000000000388

For peer review only

For peer review only - http://bmjopen.bmj.com/site/about/guidelines.xhtml

BMJ Open

Patients' views about screening for atrial fibrillation (AF): a qualitative study in primary care

Journal:	*BMJ Open*
Manuscript ID	bmjopen-2019-033061.R1
Article Type:	Original research
Date Submitted by the Author:	29-Nov-2019
Complete List of Authors:	`Lown, Mark; University of Southampton, Primary Care and Population Sciences<br>Wilcox, Christopher; University of Southampton, Primary Care and Population Sciences<br>hughes, Stephanie; University of Southampton, Primary Care and Population Sciences<br>Santer, Miriam; University of Southampton, Primary Care & Population Sciences<br>Lewith, George; University of Southampton<br>Moore, Michael; University of Southampton Medical School, Primary Care Medical Group<br>Little, Paul; University of Southampton, Primary Care and Population Science;
**Primary Subject Heading**:	Cardiovascular medicine
Secondary Subject Heading:	Qualitative research, General practice / Family practice
Keywords:	atrial fibrillation, QUALITATIVE RESEARCH, screening, general practice

SCHOLARONE™
Manuscripts

I, the Submitting Author has the right to grant and does grant on behalf of all authors of the Work (as defined in the below author licence), an exclusive licence and/or a non-exclusive licence for contributions from authors who are: i) UK Crown employees; ii) where BMJ has agreed a CC-BY licence shall apply, and/or iii) in accordance with the terms applicable for US Federal Government officers or employees acting as part of their official duties; on a worldwide, perpetual, irrevocable, royalty-free basis to BMJ Publishing Group Ltd ("BMJ") its licensees and where the relevant Journal is co-owned by BMJ to the co-owners of the Journal, to publish the Work in this journal and any other BMJ products and to exploit all rights, as set out in our licence.

The Submitting Author accepts and understands that any supply made under these terms is made by BMJ to the Submitting Author unless you are acting as an employee on behalf of your employer or a postgraduate student of an affiliated institution which is paying any applicable article publishing charge ("APC") for Open Access articles. Where the Submitting Author wishes to make the Work available on an Open Access basis (and intends to pay the relevant APC), the terms of reuse of such Open Access shall be governed by a Creative Commons licence – details of these licences and which Creative Commons licence will apply to this Work are set out in our licence referred to above.

Other than as permitted in any relevant BMJ Author's Self Archiving Policies, I confirm this Work has not been accepted for publication elsewhere, is not being considered for publication elsewhere and does not duplicate material already published. I confirm all authors consent to publication of this Work and authorise the granting of this licence.

For peer review only

Patients' views about screening for atrial fibrillation (AF): a qualitative study in primary care

Mark Lown, Christopher R Wilcox, Stephanie Hughes, Miriam Santer, George Lewith‡, Michael Moore, Paul Little,

M Lown BEng, PhD, MBBS, MRCGP, GP and Clinical Lecturer, Department of Primary Care and Population Sciences, University of Southampton, Southampton.

CR Wilcox, BSc, MBBCh, Clinical Research Fellow, NIHR Clinical Research Facility, University Hospital Southampton NHS Foundation Trust, UK, Department of Primary Care and Population Sciences, University of Southampton, Southampton

S Hughes, BSc, MSc, Senior Research Assistant, Department of Primary Care and Population Sciences, University of Southampton, Southampton.

M Santer, MBBCHir, MRCGP, PhD, Associate Professor in Primary Care Research, Department of Primary Care and Population Sciences, University of Southampton, Southampton.

G Lewith MA, MD, FRCP, MRCGP, Professor of Primary care Research, Department of Primary Care and Population Sciences, University of Southampton, Southampton.

M Moore, BM, BS, MRCP, MRCGP Professor of Primary care Research, Department of Primary Care and Population Sciences, University of Southampton, Southampton.

P Little, MBBS, BA, MD, DLSHTM, MRCP, FRCGP, FMedSci, Professor of Primary care Research, Department of Primary Care and Population Sciences, University of Southampton, Southampton.

‡Deceased

Address for correspondence:

Mark Lown Department of Primary Care and Population Sciences, Aldermoor Health Centre, Aldermoor Close, University of Southampton, Southampton SO165ST.

Email: m.lown@soton.ac.uk

For peer review only

Abstract

Objectives

There has been increased interest in screening for Atrial Fibrillation (AF) with commissioned pilot schemes, ongoing large clinical trials and the emergence of inexpensive consumer single-lead ECG devices that can be used to detect AF. This qualitative study aimed to explore patients' views and understanding of AF and AF screening in order to determine acceptability and inform future recommendations.

Setting

A single primary care practice in Hampshire, UK.

Participants

15 participants (11 female) were interviewed from primary care who had taken part in an AF screening trial. A semi-structured interview guide was used flexibly, to enable the interviewer to explore any relevant topics raised by the participants. Interviews were recorded, transcribed verbatim, and analysed using inductive thematic analysis.

Results

Participants generally had an incomplete understanding of AF and conflated it with other heart problems or with raised blood pressure. With regards to potential drawbacks from screening, some participants considered anxiety and the cost of implementation, but none acknowledged potential harms associated with screening such as side effects of anti-coagulation treatment or the risk of further investigations. The screening was generally well accepted, and participants were generally in favour of engaging with prolonged screening.

Conclusions

Our study highlights that there may be poor understanding (of both the nature of AF and potential negatives of screening) amongst patients who have been screened for AF. Further work is required to determine if resources including decision aids can address this important knowledge gap, and improve clinical informed consent for AF screening.

Trial Registration: ISRCTN: ISRCTN 17495003.

For peer review only

Strengths and limitations of this study

- A strength of this study is that all the interviews were conducted by a single researcher, thus ensuring consistency.
- The study had good representation in terms of a typical screening population aged over 65 years, although there were only four male participants and all participants were from a single GP surgery in England.
- All of the participants had taken part in the SAFETY AF screening trial and those not wishing to be screened for atrial fibrillation may have had different views.
- Participants were recruited from a single surgery and may have had similar views, not representative of the wider population. However, they were form an area of low deprivation and as they had taken part in a trial were likely to be more health literate than the wider population who may have a poorer understanding of AF.
- The participants all had a negative screen which may have affected their attitudes.

For peer review only

Introduction

Atrial fibrillation (AF) is a common heart rhythm irregularity, characterised by abnormal beating of the atria, and affects around 10% of people aged over 65 in the UK.[1] AF is associated with an increased risk of stroke which is substantially reduced by anticoagulation.[2] As a significant proportion of AF is paroxysmal and can be asymptomatic, it can often go undiagnosed: across England, it is estimated that 425,000 people are living with undiagnosed AF.[3] There has been much recent debate about screening for AF[4] by expert committees and in UK parliament. Currently, no Randomised Controlled Trial (RCT) evidence exists for systematic AF screening for stroke prevention and no country has yet implemented a systematic screening programme. However, the best available evidence does provide a strong case for screening, and the European Cardiac Society currently recommends opportunistic screening in patients aged > 65 and consideration of systematic screening in patients aged > 75 or in those at higher stroke risk.[5] The UK national screening committee is due to review its decision on AF screening imminently.

NHS England has, however, commissioned pilot AF screening schemes in pharmacies and in flu clinics to 'test the treatments and care models of tomorrow.' [6,7] There are ongoing, large RCTs to investigate the cost-effectiveness of AF screening and the outcomes are eagerly awaited.[8] We are also witnessing a shift in healthcare with increased consumerisation of medical devices and the emergence of relatively inexpensive consumer single-lead ECG devices that can be used for AF detection.[9] Consumer watches are now available with ECG capability and the potential to diagnose AF.[10] Hence, there now exists a great need to ensure patients are well informed before undertaking any form of AF screening and to understand patient attitudes towards screening for AF. There is a sparsity of qualitative data in the literature on patient beliefs and attitudes towards screening for AF and in this article, we report on an interview study that aimed to explore patient views on screening for AF.

Methods

The current qualitative study was nested within the Screening for Atrial Fibrillation using Economical and Accurate TechnologY (SAFETY) study.[11] 418 participants were recruited to the SAFETY trial from three primary care practices in the Wessex area. Individuals aged over 65 years both with and without a coded diagnosis of AF in their medical records were

For peer review only - http://bmjopen.bmj.com/site/about/guidelines.xhtml

invited by their GP to a single nurse-led screening visit in order to test the accuracy of several devices (a blood pressure meter, a single-lead device and two ECG sensing consumer devices) for the detection of AF. Research nurses explained how to use the devices and participants were able to ask questions about AF and the devices during the visit.

Data collection

For this qualitative study, a convenience sample (Participants were) of 34 of the 418 trial participants not known to have AF from a single GP surgery were invited to take part in an interview. Participants who consented to the qualitative study were approached sequentially (in order of randomisation to the main study) by telephone. Of these, 15 agreed to participate. We only invited participants who did not have AF as they would likely be more representative of a typical screening population. We did not record reasons for declining to participate. All the participants had previously been sent information leaflets as part of the trial invitation with detailed information on AF, screening for AF, and treatment options if AF were detected. Interviews were conducted by SH (a senior researcher with considerable qualitative research experience) via telephone, audio-recorded and transcribed verbatim, assigning ID numbers to preserve anonymity. A semi-structured interview guide was used flexibly, to enable the interviewer to explore any relevant topics raised by the participants (Table 1). The interview guide covered topics such as the patient's understanding of AF, views about AF screening (including benefits and drawbacks), opinions about the devices trialled (e.g. device comfort) and opinions about future use of the devices. The interviews were carried out between May 2017 and July 2017 and lasted around 15 minutes.

Data analysis

Interview transcripts were analysed using inductive thematic analysis (this approach aims to generate new theory from patterns or themes of meaning within data as there is little data in the literature on patient view on AF screening)[12]. Transcripts were read and re-read (ML, SH, CW, MS) to identify codes, which were then organised iteratively into a coding manual by ML and CW. Main themes and sub-themes were generated independently by ML and CW then reviewed and refined through further discussions within the team, which included a mix of clinicians and academics with varying degrees of experience. We assessed the data for saturation of main themes and searched for disconfirming cases (the authors reviewed

For peer review only

themes emerging during coding and were confident saturation had been reached when no new themes of interest were arising.) The sample size was appropriate and sufficient to achieve saturation[13].

Patient and public involvement

Patient and public representatives were involved in the design of the study from the funding application stage and in protocol development. All the study materials were developed with lay input.

Results

Participant characteristics

Of the 15 participants who took part in the qualitative study, four were male (26.7%) and the average age was 68 years (range = 65-73) (SD = 2.74). The index of multiple deprivation score for the participating GP practice was 18.2 and the income deprivation score affecting older people (> 60 years) index was 17.2, reflecting low levels of deprivation. All participants were fluent in English. A number of themes emerged relating to their [1] understanding of AF, [2] attitudes to screening (in general and for AF specifically), [3] attitudes to the screening devices tested during the SAFETY study, and [4] their attitudes to undergoing prolonged screening.

Understanding of atrial fibrillation

Participants were asked to describe their understanding of AF. In interpreting responses to this question, it needs to be highlighted that all participants had received an information sheet, which described AF as 'an irregular heart rhythm that can lead to blood clots forming within the heart which can come loose and cause a stroke.' Despite this, there was considerable confusion about the nature of the condition. Although the majority of participants said they were aware that AF related to a problem with the heart, many (6/15) seemed unaware that it related to heart rhythm irregularity, and few acknowledged its association with risk of stroke or developing clots (2/15).

> *"Something wrong with your heart system…of the atrium and connecting pipes"* [P15]

For peer review only

"Well, to identify a possible stroke and high blood pressure and possible stroke and heart attack." [P5]

"it's a condition that is related to the blood circulation" [P43]

"Well if the heart isn't functioning properly and this is a condition that could be picked up…if you have heart problems" [P65]

Attitudes to screening

When asked for their views regarding screening for atrial fibrillation, many of the participants stated positive opinions about health screening in general, with regards to early detection and saving money for the health service.

"I believe in screening as much as possible…anything that helps with picking up conditions, I think, is a good thing" [P72]

"Having more treatments or monitoring available…can't be a bad thing" [P122]

"Saves the health service a hell of a lot of money…any kind of health screening on the NHS system is a very good idea" [P131]

Many participants raised positive opinions regarding early detection and treatment of AF specifically, and two mentioned prevention of stroke.

"I think it's sensible to know if you've got AF, because then it's possible to have some treatment" [P39]

In response to the question "What are your thoughts about screening for atrial fibrillation": *"Well I actually think it's a good idea; there's a lot of heart conditions in my own family on my father's side, and so it's no harm to be monitored every so often, along the way"* [P53]

"I think it's an excellent idea; better to catch a condition early if you possibly can, especially if people…[are] not aware they have it…if there's medication or other things to stop it…I know there are so many other calls on the Health Service, but I know stroke and so on being so debilitating; if you can avoid any at all, that would be a good thing" [P125]

For peer review only

Most participants didn't describe any potential downsides to screening for AF. Possible negatives that were raised by participants included anxiety and cost for the health service.

"It might make you anxious, but my anxiety on that score would be counteracted by - at least I know and it's now in hand, as opposed to not knowing and then it causing a complication" [P39]

"Perhaps…it might trigger people to be too anxious about their health, perhaps if they were …that sort of person" [P125]

"I suppose the cost for the NHS but, in the long-term, if you can pick something up early and correct it, it's going to be cheaper than if it's left and then down the road they are going to need more care and intervention" [P72]

"I suspect it's probably desirable…the cost or the complexity might outweigh the benefits; I'm not completely sure on that" [P122]

Participants may have misunderstood the scope of the screening test. One participant felt reassured they had a healthy heart.

"Well I suppose it was a chance to see if my heart was healthy; also it reassured me" [P65]

Attitudes to the screening devices

Some participants stated no particular preference towards any device, and felt that all were comfortable.

"Absolutely no question of them being unpleasant…they were very unintrusive and unobjectionable" [P122]

"They were all comfortable. I could have coped with any of them…if I'd been selected to use a particular type it wouldn't have bothered me" [P72]

Others participants had mixed opinions regarding the user-friendliness of the devices, and stated a preference towards those which were the least uncomfortable, least trouble, and least time-consuming to use (although their opinion as to which device was preferred differed between participants).

For peer review only

"I think probably the one on the finger was most convenient to use. There was another one where you had to put patches on yourself...[which] I would probably have to force myself to do at home…it would just be one of those irritating things to do. There was another one…that every time you used it, you were going to have to turn on the computer, log into a site and it would upload the data onto the site... at a specific and regular time…[which would be] a bit of a faff." [P45]

"They were all fine. I think the most difficult one…where you have all the things put on you…that's the one that takes the time. But all the others seemed very, you know, quick…I think the first one was the simplest, the one with the thumbs" [P76]

"*The worst one was the blood pressure cuff which was quite uncomfortable. The one…you wore it round your [rib cage] – you weren't aware you were wearing it and I think that was the best one…The handheld device was quite comfortable but…you were holding it, so whereas wearing the thing around your chest, your hands were free to do other things…and the ECG, obviously, you had to be fully engaged with that and you couldn't do other things*" [P125]

Attitudes towards undertaking prolonged screening

Many participants stated that they would be happy to undergo prolonged screening using these (or similar) screening devices if it were recommended by their doctor.

"I just tend to follow advice with that sort of stuff…if it's because there was a need…it would be in my best interest then I would do yes" [P15]

"Even if it was uncomfortable…you would just do what you had to do really…I wouldn't have any hesitation if it was a health matter" [P125]

Others were more reserved about the idea of prolonged screening over a number of weeks or had specific concerns, for instance about the time required and the potential of screening to provoke anxiety or 'take over your life'.

"*Well I'm not sure. It depends how many weeks we're talking about…I mean I don't have any other particular issue other than the timing*" [P131]

For peer review only

"I'm probably a bit of an anxious person…so I might not be quite at ease…I'm not the sort of person that happily does something and just forgets…it tends to take over your life a bit…so if [it was] for two weeks or so, I might be a bit hesitant" [P130]

Some participants seemed unclear or doubtful about prolonged screening, i.e. they would be happy to test for AF if there was a definitive need identified by their doctor but not just if it was a matter of their age. This suggests that the test, even if they felt the test was tolerable, they may have reservations about doing this in the absence of symptoms.

"I mean if there was a valid reason behind it...if it was just oh you're getting to a certain age and we ought to look at it, I would probably…have a bit of a half-hearted effort at it…but if the doctor…[said that there] might be a problem here and it needs further investigation, then obviously I would take it quite seriously" [P45]

Discussion

In this study, participants seemed to have an incomplete understanding of AF and conflated it with other heart problems or with raised blood pressure. Patients expressed positive opinions towards screening, diagnosing AF at an early stage and the potential to save costs. Additionally, more than half of those invited for screening responded and were willing to take part in the SAFETY trial.[14] With regards to potential drawbacks from screening, some participants did consider anxiety and the cost of implementation, but none acknowledged potential harms associated with screening such as side effects of anti-coagulation treatment, the risk of further investigations, or the accuracy of the diagnostic test and potential for false positive or false negative results. The screening devices in the SAFETY trial were generally well accepted, however some participants preferred unobtrusive and more user-friendly devices. Participants were generally in favour of engaging with prolonged screening (for several weeks) although some said they would only do so if advised by a health care professional or if they had a 'health problem', and had concerns about time requirement and the potential for the testing to provoke anxiety.

Despite the fact that participants had received printed information sheets as part of the SAFETY trial (which included information on AF [including paroxysmal AF], its diagnosis, and

For peer review only

anticoagulation treatment), they still appeared to have limited understanding of AF – a finding which is consistent with recent literature: previous studies have identified that many patients with AF were not aware of the name of the condition[15] or that it led to an increased risk of stroke.[16] Although a blood pressure meter was employed as one of the screening devices, the other 3 devices used in the trial measured ECG signals and participants had a 12-Lead ECG and were informed that it was a definitive diagnostic test. Other work has found that patients were uncertain what AF was before and after out-patient cardiology clinic appointments.[17] They also had difficulty understanding why they were treated with anti-coagulation, and why treatment was recommended lifelong.[17] Older patients, in particular, may have a poor understanding of AF, which in turn may have a negative impact on their life.[18] Furthermore, a lack of knowledge may be a key barrier to accepting anti-coagulation treatment and future adherence - which is imperative for stroke risk reduction.[19]

Some participants did mention the possibility that screening might provoke anxiety, and although there is a lack of evidence of this for AF screening specifically, data does suggest that screening can induce anxiety in breast and cervical screening programs.[20-21] The literature also suggests that there is potential for psychological harm from being labelled with an unexpected diagnosis through screening for other conditions.[22] However, when directly asked about potential downsides of screening in this study, participants did not mention the potential risk of harm from treatment, or the risk of further investigations when deciding to participate, suggesting that this was not a major concern for them. This is consistent with other studies showing that many patients are unable to identify potential harms from screening tests, and of those that did, they were mostly related to the test itself, not to further testing or treatment.[23] In contrast, patients could name benefits and tended to overestimate them.[23]

Importantly, when asked for their opinions on any potential drawbacks about screening in general, participants did not mention the accuracy of the diagnostic test or the possibility for false positive and/or negative diagnoses, or discuss the potential for over-diagnosis. Again, there is no AF-specific data in the literature and a general lack of understanding of AF and treatment options could account for the lack of concerns regarding diagnostic accuracy. Other work has found many AF patients were unaware that AF could be asymptomatic and

therefore they may not be aware of paroxysmal episodes which could remain undiagnosed.[24] Patients do have worry about potential false positive results for other screening tests such as lung cancer tests.[25] There is also concern that patients may not understand the concept of over-diagnosis[20]. However, evidence suggests that older patients may be suspicious or resistant to the concept of over-detection of other conditions.[20,26] Interestingly, there is also evidence that older adults perceived overuse to have occurred when interventions were used in the absence of symptoms (excluding cancer screening), did not improve symptoms, or against their preferences.[27]

The screening devices were generally well accepted by the participants and found to be unobtrusive, but some participants did express concerns about comfort, user-friendliness and time taken to use the device. AF screening devices have been found to be well-accepted by participants in previous large-scale trials.[28,29] However, many participants stated they would be happy to undergo prolonged screening only if recommended by their doctor and some participants had specific concerns with respect to the time taken and the potential to provoke anxiety.

Practice and policy implications

There is a need to provide clear and concise information about AF, and to check patient understanding, before proceeding with any form of screening. Checklists could be used to ensure key points have been discussed and considered, and patients may require time to weigh up the risks and benefits before deciding to proceed with screening. Decision aids have been implemented for AF treatment but have usually been designed to support clinician decisions and do not explicitly engage patients.[30] Decision aids could potentially improve patient knowledge prior to screening and have been used to improve knowledge for other screening programs.[31-34] They could be used to explain the diagnostic test, conditions that could be diagnosed, quantitative information relating to diagnostic accuracy and the risks and benefits of treatment. They could also be used to promote clarification of patients' preferences about the screening and potential consequences in order to improve patient knowledge. Health care professionals could actively ask about any potential anxiety participants may have prior to screening and be prepared to discuss these.

For peer review only

Consent for screening rests on having sufficient information about the procedure and its potential downsides. Adequate information prior to screening should include the risk of bleeding with anticoagulation treatment if AF were detected. A discussion of available treatment options and lifestyle modifications before undergoing testing might also ensure the potential for future compliance if AF was indeed diagnosed. Patients should also be informed about the specificity of the screening test, and reminded that screening will not provide information on general heart function or cardiovascular risk in order to avoid false reassurance. If patients have concerns over the time and effort required for prolonged screening, perhaps discussion of individual risks and benefits before proceeding could aid decision-making.

Patients should be well- informed regarding the test accuracy and the potential for false negatives which could increase compliance if prolonged or repeated screening were offered. An understanding of the paroxysmal or asymptomatic nature of AF in patients with intermittent AF may enhance treatment adherence. As participants expressed opinions on the user-friendliness and comfort of devices, these issues should be considered whenever new devices are considered for AF screening or detection. The emergence of consumer ECG devices and wearable technology such as watches that can detect pulse irregularity and ECG sensing capability[4], will inevitably lead to an increase in asymptomatic patients presenting with suspected irregular pulses or subclinical AF, and we should ensure patients are well informed before referring for further tests (or to disregard this functionality until it has been further assessed). Furthermore, given the lack of RCT evidence for screening and the heterogeneity in stroke risk estimation data,[35] clinicians may be required to have discussions around uncertainty and over-diagnosis, particularly in asymptomatic patients who have effectively self-screened themselves for AF.

Conclusions

Our study highlights that there may be poor understanding (of both the nature of AF and potential negatives of screening) amongst patients who have been screened for AF. Further work is required to determine if resources including decision aids can address this important knowledge gap, and improve clinical informed consent for AF screening.

For peer review only

Word count 3685

Acknowledgements: The authors would like to thank all the participants who took part in this study: without them this study would not have been possible.

Contributions: ML, GL, MM, PL designed the study. SH conducted the interviews. ML & CW coded and analysed the interview data. ML, CW, SH & MS contributed to the interpretation of the data. ML drafted the manuscript and all authors contributed to the review and editing of the manuscript.

Funding: This study/project is funded by the National Institute for Health Research (NIHR) School for Primary Care Research (project reference 318). The views expressed are those of the authors and not necessarily those of the NIHR or the Department of Health and Social Care.

Competing Interests: None declared.

Ethics approval: The study complies with the declaration of Helsinki, and the protocol was approved by the London - City & East Research Ethics Committee in June 2016 (ref 16/LO/1173). Informed consent was obtained from all participants (trial registration ISRCTN: 17495003).

Data availability statement: No data are available.

References

1. Cowan C, Healicon R, Robson I, et al. The use of anticoagulants in the management of atrial fibrillation among general practices in England. *Heart* 2013; **99(16)**: 1166-72.
2. Hart RG, Pearce LA, Aguilar MI. Meta-analysis: antithrombotic therapy to prevent stroke in patients who have nonvalvular atrial fibrillation. *Ann Intern Med.* 2007; **146(12)**:857-67.
3. Reiffel JA, Atrial fibrillation and stroke: epidemiology. *Am J Med.* 2014; **127(4)**:e15-6.
4. Lown M, Moran P. Should we screen for atrial fibrillation? *BMJ.* 2019 Feb 13;364:l43.
5. Mairesse GH, Moran P, Van Gelder IC, Elsner C, Rosenqvist M, Mant J, Banerjee A, Gorenek B, Brachmann J, Varma N, Glotz de Lima G, Kalman J, Claes N, Lobban T, Lane D, Lip GYH, Boriani G; ESC Scientific Document Group. Screening for atrial fibrillation: a European Heart Rhythm Association (EHRA) consensus document endorsed by the Heart Rhythm Society (HRS), Asia Pacific Heart Rhythm Society (APHRS), and Sociedad Latinoamericana de Estimulación Cardíaca y Electrofisiología (SOLAECE). *Europace.* 2017; **19(10)**:1589-1623.

For peer review only

6. Care City launches life-saving atrial fibrillation pathway. https://nhsaccelerator.com/care-city-launches-life-saving-atrial-fibrillation-pathway/. Accessed 01/03/2019.
7. Atrial Fibrillation Check in Influenza Vaccination Programme. http://www.londonscn.nhs.uk/wp-content/uploads/2017/07/heywood-middleton-and-rochdale-ccg.pdf Accessed 01/03/2019.
8. Nuffield Department of Primary Care Health Sciences. Upcoming trial involving 120 000 patients to investigate screening for atrial fibrillation. https://www.phc.ox. ac.uk/news/new-research-to-investigate-whether-screening-can-prevent-one-in-ten-strokes.Accessed 01/03.2019.
9. Gropler MRF, Dalal AS, Van Hare GF, Silva JNA. Can smartphone wireless ECGs be used to accurately assess ECG intervals in pediatrics? A comparison of mobile health monitoring to standard 12-lead ECG. *PLoS One*. 2018;**13(9)**:e0204403.
10. Apple Watch Series 4: Proactive Health Monitor. https://www.apple.com/apple-watch-series-4/health/ accessed 01/04/2019.
11. Lown M, Yue A, Lewith G, Little P, Moore M. Screening for Atrial Fibrillation using Economical and accurate TechnologY (SAFETY)-a pilot study. *BMJ Open*. 2017;**7(1)**:e013535.
12. Braun V, Clarke V. Using Thematic Analysis in Psychology. *Qualitative Research in Psychology* 2006. **3(2)**:77-101
13. Guest G, Bruce A, Johnson L. How many interviews are enough? An experiment with data saturation and variability. *Field Methods* 2006; **18(1)**: 59-82.
14. Lown M, Yue AM, Shah BN, Corbett SJ, Lewith G, Stuart B, Garrard J, Brown M, Little P, Moore M. Screening for Atrial Fibrillation Using Economical and Accurate Technology (From the SAFETY Study). Am J Cardiol. 2018 Oct 15;122(8):1339-1344.
15. Lip GY, Kamath S, Jafri M, Mohammed A, Bareford D. Ethnic differences in patient perceptions of atrial fibrillation and anticoagulation therapy: the West Birmingham Atrial Fibrillation Project. *Stroke.* 2002; **33(1)**:238-42
16. McCabe PJ, Schad S, Hampton A, Holland DE. Knowledge and self-management behaviors of patients with recently detected atrial fibrillation. *Heart Lung.* 2008; **37(2)**:79-90.
17. Thrysoee L, Strömberg A, Brandes A, Hendriks JM. Management of newly diagnosed atrial fibrillation in an outpatient clinic setting-patient's perspectives and experiences*. J Clin Nurs.* 2018; **27(3-4)**:601-611.
18. Lindberg T, Sanmartin Berglund J, Elmståhl S, Bohman DM. Older individuals' need for knowledge and follow-up about their chronic atrial fibrillation, lifelong medical treatment and medical controls. *Scand J Caring Sci.* 2017; **31(4)**:1022-1030.
19. Salmasi S, Kwan L, MacGillivray J, Bansback N, De Vera MA, Barry AR, Harrison MJ, Andrade J, Lynd LD, Loewen P. Assessment of atrial fibrillation patients' education needs from patient and clinician perspectives: A qualitative descriptive study. *Thromb Res.* 2019;**173**:109-116.
20. Mathioudakis AG, Salakari M, Pylkkanen L, Saz-Parkinson Z, Bramesfeld A, Deandrea S, Lerda D, Neamtiu L, Pardo-Hernandez H, Solà I, Alonso-Coello P. Systematic review on women's values and preferences concerning breast cancer screening and diagnostic services. *Psychooncology*. 2019 Feb 27.
21. Patel H, Moss EL, Sherman SM. HPV primary cervical screening in England: Women's awareness and attitudes. *Psychooncology*. 2018; **27(6)**:1559-1564.
22. Cotter AR, Vuong K, Mustelin L, Yang Y, Rakhmankulova M, Barclay CJ, Harris RP. Do psychological harms result from being labelled with an unexpected diagnosis of abdominal aortic aneurysm or prostate cancer through screening? A systematic review. *BMJ Open*. 2017; **7(12)**:e017565
23. Sutkowi-Hemstreet A, Vu M, Harris R, Brewer NT, Dolor RJ, Sheridan SL. Adult Patients' Perspectives on the Benefits and Harms of Overused Screening Tests: a Qualitative Study. *J Gen Intern Med.* 2015; **30(11):**1618-26.
24. Salmasi S, De Vera MA, Barry A, Bansback N, Harrison M, Lynd LD, Loewen PS. Assessment of Condition and Medication Knowledge Gaps Among Atrial Fibrillation Patients: A Systematic Review and Meta-analysis. *Ann Pharmacother*. 2019 Mar 2.
25. Carter-Harris L, Brandzel S, Wernli KJ, Roth JA, Buist DSM. A qualitative study exploring why individuals opt out of lung cancer screening. *Fam Pract.* 2017;**34(2)**:239-244
26. Pappadis MR, Volk RJ, Krishnan S, Weller SC, Jaramillo E, Hoover DS, Giordano SH, Tan A, Sheffield KM, Housten AJ, Goodwin JS. Perceptions of overdetection of breast cancer among women 70 years of age and older in the USA: a mixed-methods analysis. *BMJ Open*. 2018**; 8(6)**:e022138.

For peer review only

27. Green AR, Tung M, Segal JB. Older Adults' Perceptions of the Causes and Consequences of Healthcare Overuse: A Qualitative Study. *J Gen Intern Med*. 2018;**33(6)**:892-897.
28. Lowres N, Krass I, Neubeck L, Redfern J, McLachlan AJ, Bennett AA, Freedman SB. Atrial fibrillation screening in pharmacies using an iPhone ECG: a qualitative review of implementation. *Int J Clin Pharm.* 2015;**37(6)**:1111-20.
29. Svennberg E, Engdahl J, Al-Khalili F, Friberg L, Frykman V, Rosenqvist M. Mass Screening for Untreated Atrial Fibrillation: The STROKESTOP Study. *Circulation*. 2015;**131(25)**:2176-84.
30. Probst MA, Noseworthy PA, Brito JP, Hess EP. Shared Decision-Making as the Future of Emergency Cardiology. *Can J Cardiol*. 2018;**34(2)**:117-124.
31. Perestelo-Perez L, Rivero-Santana A, Torres-Castaño A, Ramos-Garcia V, Alvarez-Perez Y, Gonzalez-Hernandez N, Buron A, Pignone M, Serrano-Aguilar P. Effectiveness of a decision aid for promoting colorectal cancer screening in Spain: a randomized trial. *BMC Med Inform Decis Mak.* 2019;**19(1)**:8.
32. Smith SK, Cai A, Wong M, Sousa MS, Peate M, Welsh A, Meiser B, Kaur R, Halliday J, Lewis S, Trevena L, Yanes T, Barlow-Stewart K, Barclay M. Improving women's knowledge about prenatal screening in the era of non-invasive prenatal testing for Down syndrome - development and acceptability of a low literacy decision aid. *BMC Pregnancy Childbirth*. 2018;**18(1)**:499.
33. Wang Y, Anazodo A, Logan S. Systematic review of fertility preservation patient decision aids for cancer patients. *Psychooncology*. 2019 Mar;**28(3):**459-467.
34. Ivlev I, Jerabkova S, Mishra M, Cook LA, Eden KB. Prostate Cancer Screening Patient Decision Aids: A Systematic Review and Meta-analysis. *Am J Prev Med.* 2018;**55(6)**:896-907
35. Quinn GR, Severdija ON, Chang Y, Singer DE Wide variation in reported rates of stroke across cohorts of patients with atrial fibrillation. *Circulation* 2017;**135**:208-19.

For peer review only

**Interview Schedule**
Can you describe your understanding of atrial fibrillation?
What are your views about screening for atrial fibrillation?<br>- What do you think the positives of screening are?<br>- What do you think the negatives of screening are?
What did you think about the study information given to you?
Did you have any reservations about taking part, and if so, can you tell me a bit about them?
Do you have any regrets about taking part? If so, can you tell me a bit about them?
Could you tell me a bit about the devices you tried as part of the SAFETY trial?
How comfortable did you find the devices?
How would you feel about wearing or using the devices for a few weeks for screening?
Is there anything else you'd like to share about your participation in the trial, or your thoughts about AF

Table 1 – Interview Schedule

For peer review only

Standards for Reporting Qualitative Research (SRQR)*

http://www.equator-network.org/reporting-guidelines/srqr/

Title and abstract

	Page/line no(s).
**Title** - Concise description of the nature and topic of the study Identifying the study as qualitative or indicating the approach (e.g., ethnography, grounded theory) or data collection methods (e.g., interview, focus group) is recommended	1
**Abstract** - Summary of key elements of the study using the abstract format of the intended publication; typically includes background, purpose, methods, results, and conclusions	2

Introduction

**Problem formulation** - Description and significance of the problem/phenomenon studied; review of relevant theory and empirical work; problem statement	4
**Purpose or research question** - Purpose of the study and specific objectives or questions	4

Methods

**Qualitative approach and research paradigm** - Qualitative approach (e.g., ethnography, grounded theory, case study, phenomenology, narrative research) and guiding theory if appropriate; identifying the research paradigm (e.g., postpositivist, constructivist/ interpretivist) is also recommended; rationale**	4-5
**Researcher characteristics and reflexivity** - Researchers' characteristics that may influence the research, including personal attributes, qualifications/experience, relationship with participants, assumptions, and/or presuppositions; potential or actual interaction between researchers' characteristics and the research questions, approach, methods, results, and/or transferability	5
**Context** - Setting/site and salient contextual factors; rationale**	5
**Sampling strategy** - How and why research participants, documents, or events were selected; criteria for deciding when no further sampling was necessary (e.g., sampling saturation); rationale**	5
**Ethical issues pertaining to human subjects** - Documentation of approval by an appropriate ethics review board and participant consent, or explanation for lack thereof; other confidentiality and data security issues	13
**Data collection methods** - Types of data collected; details of data collection procedures including (as appropriate) start and stop dates of data collection and analysis, iterative process, triangulation of sources/methods, and modification of procedures in response to evolving study findings; rationale**	5

For peer review only

**Data collection instruments and technologies** - Description of instruments (e.g., interview guides, questionnaires) and devices (e.g., audio recorders) used for data collection; if/how the instrument(s) changed over the course of the study	5
**Units of study** - Number and relevant characteristics of participants, documents, or events included in the study; level of participation (could be reported in results)	5
**Data processing** - Methods for processing data prior to and during analysis, including transcription, data entry, data management and security, verification of data integrity, data coding, and anonymization/de-identification of excerpts	5
**Data analysis** - Process by which inferences, themes, etc., were identified and developed, including the researchers involved in data analysis; usually references a specific paradigm or approach; rationale**	5
**Techniques to enhance trustworthiness** - Techniques to enhance trustworthiness and credibility of data analysis (e.g., member checking, audit trail, triangulation); rationale**	5

Results/findings

**Synthesis and interpretation** - Main findings (e.g., interpretations, inferences, and themes); might include development of a theory or model, or integration with prior research or theory	6-9
**Links to empirical data** - Evidence (e.g., quotes, field notes, text excerpts, photographs) to substantiate analytic findings	6-9

Discussion

**Integration with prior work, implications, transferability, and contribution(s) to the field** - Short summary of main findings; explanation of how findings and conclusions connect to, support, elaborate on, or challenge conclusions of earlier scholarship; discussion of scope of application/generalizability; identification of unique contribution(s) to scholarship in a discipline or field	10-13
**Limitations** - Trustworthiness and limitations of findings	3

Other

**Conflicts of interest** - Potential sources of influence or perceived influence on study conduct and conclusions; how these were managed	13
**Funding** - Sources of funding and other support; role of funders in data collection, interpretation, and reporting	13

*The authors created the SRQR by searching the literature to identify guidelines, reporting standards, and critical appraisal criteria for qualitative research; reviewing the reference lists of retrieved sources; and contacting experts to gain feedback. The SRQR aims to improve the transparency of all aspects of qualitative research by providing clear standards for reporting qualitative research.

For peer review only

**The rationale should briefly discuss the justification for choosing that theory, approach, method, or technique rather than other options available, the assumptions and limitations implicit in those choices, and how those choices influence study conclusions and transferability. As appropriate, the rationale for several items might be discussed together.

Reference:
O'Brien BC, Harris IB, Beckman TJ, Reed DA, Cook DA. **Standards for reporting qualitative research: a synthesis of recommendations.** *Academic Medicine*, Vol. 89, No. 9 / Sept 2014 DOI: 10.1097/ACM.0000000000000388

For peer review only

For peer review only - http://bmjopen.bmj.com/site/about/guidelines.xhtml

BMJ Open

Patients’ views about screening for atrial fibrillation (AF): a qualitative study in primary care

Journal:	*BMJ Open*
Manuscript ID	bmjopen-2019-033061.R2
Article Type:	Original research
Date Submitted by the Author:	22-Jan-2020
Complete List of Authors:	`Lown, Mark; University of Southampton, Primary Care and Population Sciences<br>Wilcox, Christopher; University of Southampton, Primary Care and Population Sciences<br>hughes, Stephanie; University of Southampton, Primary Care and Population Sciences<br>Santer, Miriam; University of Southampton, Primary Care & Population Sciences<br>Lewith, George; University of Southampton<br>Moore, Michael; University of Southampton Medical School, Primary Care Medical Group<br>Little, Paul; University of Southampton, Primary Care and Population Science;
**Primary Subject Heading**:	Cardiovascular medicine
Secondary Subject Heading:	Qualitative research, General practice / Family practice
Keywords:	atrial fibrillation, QUALITATIVE RESEARCH, screening, general practice

SCHOLARONE™ Manuscripts

I, the Submitting Author has the right to grant and does grant on behalf of all authors of the Work (as defined in the below author licence), an exclusive licence and/or a non-exclusive licence for contributions from authors who are: i) UK Crown employees; ii) where BMJ has agreed a CC-BY licence shall apply, and/or iii) in accordance with the terms applicable for US Federal Government officers or employees acting as part of their official duties; on a worldwide, perpetual, irrevocable, royalty-free basis to BMJ Publishing Group Ltd ("BMJ") its licensees and where the relevant Journal is co-owned by BMJ to the co-owners of the Journal, to publish the Work in this journal and any other BMJ products and to exploit all rights, as set out in our licence.

The Submitting Author accepts and understands that any supply made under these terms is made by BMJ to the Submitting Author unless you are acting as an employee on behalf of your employer or a postgraduate student of an affiliated institution which is paying any applicable article publishing charge ("APC") for Open Access articles. Where the Submitting Author wishes to make the Work available on an Open Access basis (and intends to pay the relevant APC), the terms of reuse of such Open Access shall be governed by a Creative Commons licence – details of these licences and which Creative Commons licence will apply to this Work are set out in our licence referred to above.

Other than as permitted in any relevant BMJ Author's Self Archiving Policies, I confirm this Work has not been accepted for publication elsewhere, is not being considered for publication elsewhere and does not duplicate material already published. I confirm all authors consent to publication of this Work and authorise the granting of this licence.

For peer review only

Patients' views about screening for atrial fibrillation (AF): a qualitative study in primary care

Mark Lown, Christopher R Wilcox, Stephanie Hughes, Miriam Santer, George Lewith‡, Michael Moore, Paul Little,

M Lown BEng, PhD, MBBS, MRCGP, GP and Clinical Lecturer, Department of Primary Care and Population Sciences, University of Southampton, Southampton.

CR Wilcox, BSc, MBBCh, Clinical Research Fellow, NIHR Clinical Research Facility, University Hospital Southampton NHS Foundation Trust, UK, Department of Primary Care and Population Sciences, University of Southampton, Southampton

S Hughes, BSc, MSc, Senior Research Assistant, Department of Primary Care and Population Sciences, University of Southampton, Southampton.

M Santer, MBBCHir, MRCGP, PhD, Associate Professor in Primary Care Research, Department of Primary Care and Population Sciences, University of Southampton, Southampton.

G Lewith MA, MD, FRCP, MRCGP, Professor of Primary care Research, Department of Primary Care and Population Sciences, University of Southampton, Southampton.

M Moore, BM, BS, MRCP, MRCGP Professor of Primary care Research, Department of Primary Care and Population Sciences, University of Southampton, Southampton.

P Little, MBBS, BA, MD, DLSHTM, MRCP, FRCGP, FMedSci, Professor of Primary care Research, Department of Primary Care and Population Sciences, University of Southampton, Southampton.

‡Deceased

Address for correspondence:

Mark Lown Department of Primary Care and Population Sciences, Aldermoor Health Centre, Aldermoor Close, University of Southampton, Southampton SO165ST.

Email: m.lown@soton.ac.uk

For peer review only

Abstract

Objectives

There has been increased interest in screening for Atrial Fibrillation (AF) with commissioned pilot schemes, ongoing large clinical trials and the emergence of inexpensive consumer single-lead ECG devices that can be used to detect AF. This qualitative study aimed to explore patients' views and understanding of AF and AF screening in order to determine acceptability and inform future recommendations.

Setting

A single primary care practice in Hampshire, UK.

Participants

15 participants (11 female) were interviewed from primary care who had taken part in an AF screening trial. A semi-structured interview guide was used flexibly, to enable the interviewer to explore any relevant topics raised by the participants. Interviews were recorded, transcribed verbatim, and analysed using inductive thematic analysis.

Results

Participants generally had an incomplete understanding of AF and conflated it with other heart problems or with raised blood pressure. With regards to potential drawbacks from screening, some participants considered anxiety and the cost of implementation, but none acknowledged potential harms associated with screening such as side effects of anti-coagulation treatment or the risk of further investigations. The screening was generally well accepted, and participants were generally in favour of engaging with prolonged screening.

Conclusions

Our study highlights that there may be poor understanding (of both the nature of AF and potential negatives of screening) amongst patients who have been screened for AF. Further work is required to determine if resources including decision aids can address this important knowledge gap, and improve clinical informed consent for AF screening.

Trial Registration: ISRCTN: ISRCTN 17495003.

For peer review only

Strengths and limitations of this study

- A strength of this study is that all the interviews were conducted by a single researcher, thus ensuring consistency.
- The study had good representation in terms of a typical screening population aged over 65 years although the high proportion of female participants may have affected the results.
- All of the participants had taken part in the SAFETY AF screening trial and those not wishing to be screened for atrial fibrillation may have had different views.
- Participants were recruited from a single surgery and may have had similar views, not representative of the wider population. However, they were form an area of low deprivation and as they had taken part in a trial were likely to be more health literate than the wider population who may have a poorer understanding of AF, although we did not record individual demographic data.
- The participants all had a negative screen which may have affected their attitudes.

For peer review only

Introduction

Atrial fibrillation (AF) is a common arrhythmia affecting around 10% of people aged over 65 in the UK[1] and is associated with an increased risk of stroke which is substantially reduced by anticoagulation.[2] Across England, it is estimated that 425,000 people are living with undiagnosed AF[3] and there has been much recent debate about screening for AF.[4] The European Cardiac Society currently recommends opportunistic screening in patients aged > 65 and consideration of systematic screening in patients aged > 75 or in those at higher stroke risk.[5] No country has yet implemented a systematic screening programme although NHS England has commissioned pilot AF screening schemes in pharmacies and in flu clinics to 'test the treatments and care models of tomorrow.[6,7]

There are ongoing, large RCTs to investigate the cost-effectiveness of AF screening and the outcomes are eagerly awaited.[8] We are also witnessing a shift in healthcare with increased consumerisation of medical devices and the emergence of relatively inexpensive consumer single-lead ECG devices and watches that can be used for AF detection.[9,10] Hence, there now exists a great need to ensure patients are well informed before undertaking any form of AF screening and to understand patient attitudes towards screening for AF. In this article, we report on an interview study that aimed to explore patient views on screening for AF.

Methods

The current qualitative study was nested within the Screening for Atrial Fibrillation using Economical and Accurate TechnologY (SAFETY) study.[11] 418 participants were recruited to the SAFETY trial from three primary care practices in the Wessex area. Individuals aged over 65 years both with and without a coded diagnosis of AF in their medical records were invited by their GP to a single nurse-led screening visit in order to test the accuracy of several devices (a blood pressure meter, a single-lead device and two ECG sensing consumer devices) for the detection of AF. Research nurses explained how to use the devices and participants were able to ask questions about AF and the devices during the visit.

Data collection

For this qualitative study, a convenience sample (Participants were) of 34 of the 418 trial participants not known to have AF from a single GP surgery were invited to take part in an

For peer review only

interview. Participants who consented to the qualitative study were approached sequentially (in order of randomisation to the main study) by telephone (from the first recruiting site of 3 which all had low levels of deprivation). Of these, 15 agreed to participate. We only invited participants who did not have AF as they would likely be more representative of a typical screening population. We did not record reasons for declining to participate. All the participants had previously been sent information leaflets as part of the trial invitation with detailed information on AF, screening for AF, and treatment options if AF were detected. Interviews were conducted by SH (a senior researcher with considerable qualitative research experience) via telephone, audio-recorded and transcribed verbatim, assigning ID numbers to preserve anonymity. A semi-structured interview guide was used flexibly, to enable the interviewer to explore any relevant topics raised by the participants (Table 1). The interview guide covered topics such as the patient's understanding of AF, views about AF screening (including benefits and drawbacks), opinions about the devices trialled (e.g. device comfort) and opinions about future use of the devices. The interviews were carried out between May 2017 and July 2017 and lasted around 15 minutes.

Data analysis

Interview transcripts were analysed using inductive thematic analysis (as we had little prior data or predetermined theory on AF.)[12] Transcripts were read and re-read (ML, SH, CW, MS) to identify codes, which were then organised iteratively into a coding manual by ML and CW. Main themes and sub-themes were generated independently by ML and CW then reviewed and refined through further discussions within the team, which included a mix of clinicians and academics with varying degrees of experience. We assessed the data for saturation of main themes and searched for disconfirming cases (the authors reviewed themes emerging during coding and were confident saturation had been reached when no new themes of interest were arising.) The sample size was appropriate and sufficient to achieve saturation[13].

Patient and public involvement

Patient and public representatives were involved in the design of the study from the funding application stage and in protocol development. All the study materials were developed with lay input.

For peer review only

Results

Participant characteristics

Of the 15 participants who took part in the qualitative study, four were male (26.7%) and the average age was 68 years (range = 65-73) (SD = 2.74). The average age of the participants in the SAFETY trial was 73.9 years and 43% were female. The index of multiple deprivation score for the participating GP practice was 17.7 (2nd quintile in England (from low levels of deprivation to high)) and the income deprivation score affecting older people (> 60 years) index was 17.3 (middle quintile). All participants were fluent in English. A number of themes emerged relating to their [1] understanding of AF, [2] attitudes to screening (in general and for AF specifically), [3] attitudes to the screening devices tested during the SAFETY study, and [4] their attitudes to undergoing prolonged screening.

Understanding of atrial fibrillation

Participants were asked to describe their understanding of AF. In interpreting responses to this question, it needs to be highlighted that all participants had received an information sheet, which described AF as 'an irregular heart rhythm that can lead to blood clots forming within the heart which can come loose and cause a stroke.' Despite this, there was considerable confusion about the nature of the condition. Although the majority of participants said they were aware that AF related to a problem with the heart, many (6/15) seemed unaware that it related to heart rhythm irregularity, and few acknowledged its association with risk of stroke or developing clots (2/15).

> *"Something wrong with your heart system…of the atrium and connecting pipes"* [P15]

> *"Well, to identify a possible stroke and high blood pressure and possible stroke and heart attack."* [P5]

> *"it's a condition that is related to the blood circulation"* [P43]

> *"Well if the heart isn't functioning properly and this is a condition that could be picked up…if you have heart problems"* [P65]

Attitudes to screening

For peer review only

When asked for their views regarding screening for atrial fibrillation, many of the participants stated positive opinions about health screening in general, with regards to early detection and saving money for the health service.

> *"I believe in screening as much as possible…anything that helps with picking up conditions, I think, is a good thing"* [P72]

> *"Having more treatments or monitoring available…can't be a bad thing"* [P122]

> *"Saves the health service a hell of a lot of money…any kind of health screening on the NHS system is a very good idea"* [P131]

Many participants raised positive opinions regarding early detection and treatment of AF specifically, and two mentioned prevention of stroke.

> *"I think it's sensible to know if you've got AF, because then it's possible to have some treatment"* [P39]

> In response to the question "What are your thoughts about screening for atrial fibrillation": *"Well I actually think it's a good idea; there's a lot of heart conditions in my own family on my father's side, and so it's no harm to be monitored every so often, along the way"* [P53]

> *"I think it's an excellent idea; better to catch a condition early if you possibly can, especially if people…[are] not aware they have it…if there's medication or other things to stop it…I know there are so many other calls on the Health Service, but I know stroke and so on being so debilitating; if you can avoid any at all, that would be a good thing"* [P125]

Most participants didn't describe any potential downsides to screening for AF. Possible negatives that were raised by participants included anxiety and cost for the health service.

> *"It might make you anxious, but my anxiety on that score would be counteracted by - at least I know and it's now in hand, as opposed to not knowing and then it causing a complication"* [P39]

For peer review only

"Perhaps…it might trigger people to be too anxious about their health, perhaps if they were …that sort of person" [P125]

"I suppose the cost for the NHS but, in the long-term, if you can pick something up early and correct it, it's going to be cheaper than if it's left and then down the road they are going to need more care and intervention" [P72]

"I suspect it's probably desirable…the cost or the complexity might outweigh the benefits; I'm not completely sure on that" [P122]

Participants may have misunderstood the scope of the screening test. One participant felt reassured they had a healthy heart.

"Well I suppose it was a chance to see if my heart was healthy; also it reassured me" [P65]

Attitudes to the screening devices

Some participants stated no particular preference towards any device, and felt that all were comfortable.

"Absolutely no question of them being unpleasant…they were very unintrusive and unobjectionable" [P122]

"They were all comfortable. I could have coped with any of them…if I'd been selected to use a particular type it wouldn't have bothered me" [P72]

Others participants had mixed opinions regarding the user-friendliness of the devices, and stated a preference towards those which were the least uncomfortable, least trouble, and least time-consuming to use (although their opinion as to which device was preferred differed between participants).

"I think probably the one on the finger was most convenient to use. There was another one where you had to put patches on yourself...[which] I would probably have to force myself to do at home…it would just be one of those irritating things to do. There was another one…that every time you used it, you were going to have to turn on the computer, log into a site and it would upload the data onto the site... at a specific and regular time…[which would be] a bit of a faff." [P45]

For peer review only

"*They were all fine. I think the most difficult one…where you have all the things put on you…that's the one that takes the time. But all the others seemed very, you know, quick…I think the first one was the simplest, the one with the thumbs*" [P76]

"*The worst one was the blood pressure cuff which was quite uncomfortable. The one…you wore it round your [rib cage] – you weren't aware you were wearing it and I think that was the best one…The handheld device was quite comfortable but…you were holding it, so whereas wearing the thing around your chest, your hands were free to do other things…and the ECG, obviously, you had to be fully engaged with that and you couldn't do other things*" [P125]

Attitudes towards undertaking prolonged screening

Many participants stated that they would be happy to undergo prolonged screening using these (or similar) screening devices if it were recommended by their doctor.

"I just tend to follow advice with that sort of stuff…if it's because there was a need…it would be in my best interest then I would do yes" [P15]

"Even if it was uncomfortable…you would just do what you had to do really…I wouldn't have any hesitation if it was a health matter" [P125]

Others were more reserved about the idea of prolonged screening over a number of weeks or had specific concerns, for instance about the time required and the potential of screening to provoke anxiety or 'take over your life'.

"*Well I'm not sure. It depends how many weeks we're talking about…I mean I don't have any other particular issue other than the timing*" [P131]

"I'm probably a bit of an anxious person…so I might not be quite at ease…I'm not the sort of person that happily does something and just forgets…it tends to take over your life a bit…so if [it was] for two weeks or so, I might be a bit hesitant" [P130]

Some participants seemed unclear or doubtful about prolonged screening, i.e. they would be happy to test for AF if there was a definitive need identified by their doctor but not just if it was a matter of their age. This suggests that the test, even if they felt the test was tolerable, they may have reservations about doing this in the absence of symptoms.

For peer review only

"I mean if there was a valid reason behind it...if it was just oh you're getting to a certain age and we ought to look at it, I would probably…have a bit of a half-hearted effort at it…but if the doctor…[said that there] might be a problem here and it needs further investigation, then obviously I would take it quite seriously" [P45]

Discussion

Patients expressed positive opinions towards screening for AF at an early stage and the potential to save costs. Additionally, more than half of those invited for screening responded and were willing to take part in the SAFETY trial.[14] The participants seemed to have an incomplete understanding of AF (despite receiving printed information sheets about AF, paroxysmal AF and anticoagulation treatment) and conflated it with other heart problems or with raised blood pressure. Although a sphygmomanometer device was employed as one of the screening devices, the other devices used in the trial measured ECG signals and participants were informed that the 12-Lead ECG they had was a definitive diagnostic test.

Previous studies have identified that many patients with AF were not aware of the name of the condition[15] or that it led to an increased risk of stroke.[16] Other work has found that patients were uncertain what AF was before and after out-patient cardiology clinic appointments.[17] They also had difficulty understanding why they were treated with anti-coagulation, and why treatment was recommended lifelong.[17] Older patients, in particular, may have a poor understanding of AF[18]. This may be a key barrier to accepting anti-coagulation treatment and to future treatment adherence, which is imperative for stroke risk reduction.[19] Other work has found many patients were unaware that AF could be asymptomatic and therefore they may not be aware of paroxysmal episodes which could remain undiagnosed.[20]

Some participants in our study mentioned that screening might provoke anxiety, and data does suggest that screening can induce anxiety in other screening programmes including breast and cervical screening[21-22] and the potential for psychological harm from being labelled with an unexpected diagnosis.[23] However, when directly asked about potential

downsides of screening in this study, participants did not mention the potential risk of harm from anti-coagulation treatment, or the risk of further investigations when deciding to participate, suggesting that this was not a major concern for them. This is consistent with other studies showing that many patients are unable to identify potential harms from other screening tests, and of those that did, they were mostly related to the test itself, not to further testing or treatment.[24] In contrast, patients could name benefits and tended to overestimate them.[23]

Importantly, when asked for their opinions on any potential drawbacks, participants did not mention the accuracy of the diagnostic test or the potential for over-diagnosis. Patients do worry about potential false positive results for other screening tests such as lung cancer tests.[25] There is also concern that patients may not understand the concept of over-diagnosis[20]. However, evidence suggests that older patients may be suspicious or resistant to the concept of over-detection of other conditions.[20,26] Interestingly, there is also evidence that older adults perceived overuse to have occurred when interventions were used in the absence of symptoms (excluding cancer screening), did not improve symptoms, or against their preferences.[27]

The screening devices were generally well accepted by the participants and found to be unobtrusive, but some participants expressed concerns about comfort, user-friendliness and time taken to use the device. AF screening devices have been found to be well-accepted by participants in previous large-scale trials.[28,29] However, many participants in our study stated they would be happy to undergo prolonged screening only if recommended by their doctor and some participants had specific concerns with respect to the time taken.

Patients who chose not to participate in the trial may have had different views with respect to the usability and acceptance of the devices or may have chosen not to participate due to concerns over anticoagulation treatment or had greater anxiety regarding the screening process. Participants from differing areas and backgrounds may also have expressed markedly different views. We were unable to explore the views of screen positive patients.

For peer review only

Practice and policy implications

There is a need to provide clear and concise information about AF, and to check patient understanding, before proceeding with any form of screening. Checklists could be used to ensure key points have been discussed and considered, and patients may require time to weigh up the risks and benefits before deciding to proceed with screening. Decision aids have been implemented for AF treatment but have usually been designed to support clinician decisions and do not explicitly engage patients.[30] Decision aids could potentially improve patient knowledge prior to screening and have been used to improve knowledge for other screening programs.[31-34] They could be used to explain the diagnostic test, conditions that could be diagnosed, quantitative information relating to diagnostic accuracy and the risks and benefits of treatment. They could also be used to promote clarification of patients' preferences about the screening and potential consequences in order to improve patient knowledge. Health care professionals could actively ask about any potential anxiety participants may have prior to screening and be prepared to discuss these.

Adequate information prior to screening should include the risk of bleeding with anticoagulation treatment if AF were detected. A discussion of available treatment options and lifestyle modifications before undergoing testing might also ensure the potential for future treatment adherence if AF was diagnosed. Patients should also be informed about the specificity of the screening test, and reminded that screening will not provide information on general heart function or cardiovascular risk in order to avoid false reassurance.

Patients should be well-informed regarding the test accuracy and the potential to miss paroxysmal AF which could increase compliance if prolonged or repeated screening were offered. An understanding of the paroxysmal and asymptomatic nature of AF in patients with intermittent AF may enhance treatment adherence. The emergence of consumer ECG devices and watches that can detect AF[4] will inevitably lead to an increase in asymptomatic patients presenting with suspected irregular pulses or subclinical AF, and we should ensure patients are well informed before referring for further tests (or to disregard this functionality until it has been further assessed). Furthermore, given the lack of RCT evidence for screening and the heterogeneity in stroke risk estimation data,[35] clinicians may be

For peer review only

required to have discussions around uncertainty and over-diagnosis, particularly in asymptomatic patients who have effectively self-screened themselves for AF.

Conclusions

Our study highlights that, even amongst patients who have been screened for AF, there may be poor understanding of both the nature of AF and potential negatives of screening. Further work is required to determine if resources including decision aids can address this important knowledge gap, and improve clinical informed consent for AF screening.

Word count 3181

Acknowledgements: The authors would like to thank all the participants who took part in this study: without them this study would not have been possible.

Contributions: ML, GL, MM, PL designed the study. SH conducted the interviews. ML & CW coded and analysed the interview data. ML, CW, SH & MS contributed to the interpretation of the data. ML drafted the manuscript and all authors contributed to the review and editing of the manuscript.

Funding: This study/project is funded by the National Institute for Health Research (NIHR) School for Primary Care Research (project reference 318). The views expressed are those of the authors and not necessarily those of the NIHR or the Department of Health and Social Care.

Competing Interests: None declared.

Ethics approval: The study complies with the declaration of Helsinki, and the protocol was approved by the London - City & East Research Ethics Committee in June 2016 (ref 16/LO/1173). Informed consent was obtained from all participants (trial registration ISRCTN: 17495003).

Data availability statement: No data are available.

For peer review only

References

1. Cowan C, Healicon R, Robson I, et al. The use of anticoagulants in the management of atrial fibrillation among general practices in England. *Heart* 2013; **99(16)**: 1166-72.
2. Hart RG, Pearce LA, Aguilar MI. Meta-analysis: antithrombotic therapy to prevent stroke in patients who have nonvalvular atrial fibrillation. *Ann Intern Med.* 2007; **146(12)**:857-67.
3. Reiffel JA, Atrial fibrillation and stroke: epidemiology. *Am J Med.* 2014; **127(4)**:e15-6.
4. Lown M, Moran P. Should we screen for atrial fibrillation? *BMJ.* 2019 Feb 13;364:l43.
5. Mairesse GH, Moran P, Van Gelder IC, Elsner C, Rosenqvist M, Mant J, Banerjee A, Gorenek B, Brachmann J, Varma N, Glotz de Lima G, Kalman J, Claes N, Lobban T, Lane D, Lip GYH, Boriani G; ESC Scientific Document Group. Screening for atrial fibrillation: a European Heart Rhythm Association (EHRA) consensus document endorsed by the Heart Rhythm Society (HRS), Asia Pacific Heart Rhythm Society (APHRS), and Sociedad Latinoamericana de Estimulación Cardíaca y Electrofisiología (SOLAECE). *Europace.* 2017; **19(10)**:1589-1623.
6. Care City launches life-saving atrial fibrillation pathway. https://nhsaccelerator.com/care-city-launches-life-saving-atrial-fibrillation-pathway/. Accessed 01/03/2019.
7. Atrial Fibrillation Check in Influenza Vaccination Programme. http://www.londonscn.nhs.uk/wp-content/uploads/2017/07/heywood-middleton-and-rochdale-ccg.pdf Accessed 01/03/2019.
8. Nuffield Department of Primary Care Health Sciences. Upcoming trial involving 120 000 patients to investigate screening for atrial fibrillation. https://www.phc.ox. ac.uk/news/new-research-to-investigate-whether-screening-can-prevent-one-in-ten-strokes.Accessed 01/03.2019.
9. Gropler MRF, Dalal AS, Van Hare GF, Silva JNA. Can smartphone wireless ECGs be used to accurately assess ECG intervals in pediatrics? A comparison of mobile health monitoring to standard 12-lead ECG. *PLoS One*. 2018;**13(9)**:e0204403.
10. Apple Watch Series 4: Proactive Health Monitor. https://www.apple.com/apple-watch-series-4/health/ accessed 01/04/2019.
11. Lown M, Yue A, Lewith G, Little P, Moore M. Screening for Atrial Fibrillation using Economical and accurate TechnologY (SAFETY)-a pilot study. *BMJ Open*. 2017;**7(1)**:e013535.
12. Braun V, Clarke V. Using Thematic Analysis in Psychology. *Qualitative Research in Psychology* 2006. **3(2)**:77-101
13. Guest G, Bruce A, Johnson L. How many interviews are enough? An experiment with data saturation and variability. *Field Methods* 2006; **18(1)**: 59-82.
14. Lown M, Yue AM, Shah BN, Corbett SJ, Lewith G, Stuart B, Garrard J, Brown M, Little P, Moore M. Screening for Atrial Fibrillation Using Economical and Accurate Technology (From the SAFETY Study). Am J Cardiol. 2018 Oct 15;122(8):1339-1344.
15. Lip GY, Kamath S, Jafri M, Mohammed A, Bareford D. Ethnic differences in patient perceptions of atrial fibrillation and anticoagulation therapy: the West Birmingham Atrial Fibrillation Project. *Stroke.* 2002; **33(1)**:238-42
16. McCabe PJ, Schad S, Hampton A, Holland DE. Knowledge and self-management behaviors of patients with recently detected atrial fibrillation. *Heart Lung.* 2008; **37(2)**:79-90.
17. Thrysoee L, Strömberg A, Brandes A, Hendriks JM. Management of newly diagnosed atrial fibrillation in an outpatient clinic setting-patient's perspectives and experiences*. J Clin Nurs.* 2018; **27(3-4)**:601-611.
18. Lindberg T, Sanmartin Berglund J, Elmståhl S, Bohman DM. Older individuals' need for knowledge and follow-up about their chronic atrial fibrillation, lifelong medical treatment and medical controls. *Scand J Caring Sci.* 2017; **31(4)**:1022-1030.
19. Salmasi S, Kwan L, MacGillivray J, Bansback N, De Vera MA, Barry AR, Harrison MJ, Andrade J, Lynd LD, Loewen P. Assessment of atrial fibrillation patients' education needs from patient and clinician perspectives: A qualitative descriptive study. *Thromb Res.* 2019;**173**:109-116.
20. Salmasi S, De Vera MA, Barry A, Bansback N, Harrison M, Lynd LD, Loewen PS. Assessment of Condition and Medication Knowledge Gaps Among Atrial Fibrillation Patients: A Systematic Review and Meta-analysis. *Ann Pharmacother*. 2019 Mar 2.

For peer review only

21. Mathioudakis AG, Salakari M, Pylkkanen L, Saz-Parkinson Z, Bramesfeld A, Deandrea S, Lerda D, Neamtiu L, Pardo-Hernandez H, Solà I, Alonso-Coello P. Systematic review on women's values and preferences concerning breast cancer screening and diagnostic services. *Psychooncology*. 2019 Feb 27.
22. Patel H, Moss EL, Sherman SM. HPV primary cervical screening in England: Women's awareness and attitudes. *Psychooncology*. 2018; **27(6)**:1559-1564.
23. Cotter AR, Vuong K, Mustelin L, Yang Y, Rakhmankulova M, Barclay CJ, Harris RP. Do psychological harms result from being labelled with an unexpected diagnosis of abdominal aortic aneurysm or prostate cancer through screening? A systematic review. *BMJ Open*. 2017; **7(12)**:e017565
24. Sutkowi-Hemstreet A, Vu M, Harris R, Brewer NT, Dolor RJ, Sheridan SL. Adult Patients' Perspectives on the Benefits and Harms of Overused Screening Tests: a Qualitative Study. *J Gen Intern Med.* 2015; **30(11):**1618-26.
25. Carter-Harris L, Brandzel S, Wernli KJ, Roth JA, Buist DSM. A qualitative study exploring why individuals opt out of lung cancer screening. *Fam Pract.* 2017;**34(2)**:239-244
26. Pappadis MR, Volk RJ, Krishnan S, Weller SC, Jaramillo E, Hoover DS, Giordano SH, Tan A, Sheffield KM, Housten AJ, Goodwin JS. Perceptions of overdetection of breast cancer among women 70 years of age and older in the USA: a mixed-methods analysis. *BMJ Open*. 2018**; 8(6)**:e022138.
27. Green AR, Tung M, Segal JB. Older Adults' Perceptions of the Causes and Consequences of Healthcare Overuse: A Qualitative Study. *J Gen Intern Med*. 2018;**33(6)**:892-897.
28. Lowres N, Krass I, Neubeck L, Redfern J, McLachlan AJ, Bennett AA, Freedman SB. Atrial fibrillation screening in pharmacies using an iPhone ECG: a qualitative review of implementation. *Int J Clin Pharm.* 2015;**37(6)**:1111-20.
29. Svennberg E, Engdahl J, Al-Khalili F, Friberg L, Frykman V, Rosenqvist M. Mass Screening for Untreated Atrial Fibrillation: The STROKESTOP Study. *Circulation*. 2015;**131(25)**:2176-84.
30. Probst MA, Noseworthy PA, Brito JP, Hess EP. Shared Decision-Making as the Future of Emergency Cardiology. *Can J Cardiol*. 2018;**34(2)**:117-124.
31. Perestelo-Perez L, Rivero-Santana A, Torres-Castaño A, Ramos-Garcia V, Alvarez-Perez Y, Gonzalez-Hernandez N, Buron A, Pignone M, Serrano-Aguilar P. Effectiveness of a decision aid for promoting colorectal cancer screening in Spain: a randomized trial. *BMC Med Inform Decis Mak.* 2019;**19(1)**:8.
32. Smith SK, Cai A, Wong M, Sousa MS, Peate M, Welsh A, Meiser B, Kaur R, Halliday J, Lewis S, Trevena L, Yanes T, Barlow-Stewart K, Barclay M. Improving women's knowledge about prenatal screening in the era of non-invasive prenatal testing for Down syndrome - development and acceptability of a low literacy decision aid. *BMC Pregnancy Childbirth*. 2018;**18(1)**:499.
33. Wang Y, Anazodo A, Logan S. Systematic review of fertility preservation patient decision aids for cancer patients. *Psychooncology*. 2019 Mar;**28(3):**459-467.
34. Ivlev I, Jerabkova S, Mishra M, Cook LA, Eden KB. Prostate Cancer Screening Patient Decision Aids: A Systematic Review and Meta-analysis. *Am J Prev Med.* 2018;**55(6)**:896-907
35. Quinn GR, Severdija ON, Chang Y, Singer DE Wide variation in reported rates of stroke across cohorts of patients with atrial fibrillation. *Circulation* 2017;**135**:208-19.

For peer review only

Interview Schedule
Can you describe your understanding of atrial fibrillation?
What are your views about screening for atrial fibrillation?<br>- What do you think the positives of screening are?<br>- What do you think the negatives of screening are?
What did you think about the study information given to you?
Did you have any reservations about taking part, and if so, can you tell me a bit about them?
Do you have any regrets about taking part? If so, can you tell me a bit about them?
Could you tell me a bit about the devices you tried as part of the SAFETY trial?
How comfortable did you find the devices?
How would you feel about wearing or using the devices for a few weeks for screening?
Is there anything else you'd like to share about your participation in the trial, or your thoughts about AF

Table 1 – Interview Schedule

For peer review only

Standards for Reporting Qualitative Research (SRQR)*

http://www.equator-network.org/reporting-guidelines/srqr/

Title and abstract

	Page/line no(s).
**Title** - Concise description of the nature and topic of the study Identifying the study as qualitative or indicating the approach (e.g., ethnography, grounded theory) or data collection methods (e.g., interview, focus group) is recommended	1
**Abstract** - Summary of key elements of the study using the abstract format of the intended publication; typically includes background, purpose, methods, results, and conclusions	2

Introduction

**Problem formulation** - Description and significance of the problem/phenomenon studied; review of relevant theory and empirical work; problem statement	4
**Purpose or research question** - Purpose of the study and specific objectives or questions	4

Methods

**Qualitative approach and research paradigm** - Qualitative approach (e.g., ethnography, grounded theory, case study, phenomenology, narrative research) and guiding theory if appropriate; identifying the research paradigm (e.g., postpositivist, constructivist/ interpretivist) is also recommended; rationale**	4-5
**Researcher characteristics and reflexivity** - Researchers' characteristics that may influence the research, including personal attributes, qualifications/experience, relationship with participants, assumptions, and/or presuppositions; potential or actual interaction between researchers' characteristics and the research questions, approach, methods, results, and/or transferability	5
**Context** - Setting/site and salient contextual factors; rationale**	5
**Sampling strategy** - How and why research participants, documents, or events were selected; criteria for deciding when no further sampling was necessary (e.g., sampling saturation); rationale**	5
**Ethical issues pertaining to human subjects** - Documentation of approval by an appropriate ethics review board and participant consent, or explanation for lack thereof; other confidentiality and data security issues	13
**Data collection methods** - Types of data collected; details of data collection procedures including (as appropriate) start and stop dates of data collection and analysis, iterative process, triangulation of sources/methods, and modification of procedures in response to evolving study findings; rationale**	5

For peer review only - http://bmjopen.bmj.com/site/about/guidelines.xhtml

**Data collection instruments and technologies** - Description of instruments (e.g., interview guides, questionnaires) and devices (e.g., audio recorders) used for data collection; if/how the instrument(s) changed over the course of the study	5
**Units of study** - Number and relevant characteristics of participants, documents, or events included in the study; level of participation (could be reported in results)	5
**Data processing** - Methods for processing data prior to and during analysis, including transcription, data entry, data management and security, verification of data integrity, data coding, and anonymization/de-identification of excerpts	5
**Data analysis** - Process by which inferences, themes, etc., were identified and developed, including the researchers involved in data analysis; usually references a specific paradigm or approach; rationale**	5
**Techniques to enhance trustworthiness** - Techniques to enhance trustworthiness and credibility of data analysis (e.g., member checking, audit trail, triangulation); rationale**	5

Results/findings

**Synthesis and interpretation** - Main findings (e.g., interpretations, inferences, and themes); might include development of a theory or model, or integration with prior research or theory	6-9
**Links to empirical data** - Evidence (e.g., quotes, field notes, text excerpts, photographs) to substantiate analytic findings	6-9

Discussion

**Integration with prior work, implications, transferability, and contribution(s) to the field** - Short summary of main findings; explanation of how findings and conclusions connect to, support, elaborate on, or challenge conclusions of earlier scholarship; discussion of scope of application/generalizability; identification of unique contribution(s) to scholarship in a discipline or field	10-13
**Limitations** - Trustworthiness and limitations of findings	3

Other

**Conflicts of interest** - Potential sources of influence or perceived influence on study conduct and conclusions; how these were managed	13
**Funding** - Sources of funding and other support; role of funders in data collection, interpretation, and reporting	13

*The authors created the SRQR by searching the literature to identify guidelines, reporting standards, and critical appraisal criteria for qualitative research; reviewing the reference lists of retrieved sources; and contacting experts to gain feedback. The SRQR aims to improve the transparency of all aspects of qualitative research by providing clear standards for reporting qualitative research.

For peer review only

For peer review only - http://bmjopen.bmj.com/site/about/guidelines.xhtml

**The rationale should briefly discuss the justification for choosing that theory, approach, method, or technique rather than other options available, the assumptions and limitations implicit in those choices, and how those choices influence study conclusions and transferability. As appropriate, the rationale for several items might be discussed together.

Reference:

O'Brien BC, Harris IB, Beckman TJ, Reed DA, Cook DA. **Standards for reporting qualitative research: a synthesis of recommendations.** *Academic Medicine*, Vol. 89, No. 9 / Sept 2014 DOI: 10.1097/ACM.0000000000000388

For peer review only

For peer review only - http://bmjopen.bmj.com/site/about/guidelines.xhtml

Patients' views about screening for atrial fibrillation (AF): a qualitative study in primary care

Journal:	*BMJ Open*
Manuscript ID	bmjopen-2019-033061.R3
Article Type:	Original research
Date Submitted by the Author:	13-Feb-2020

Complete List of Authors:	`Lown, Mark; University of Southampton, Primary Care and Population Sciences<br>Wilcox, Christopher; University of Southampton, Primary Care and Population Sciences<br>hughes, Stephanie; University of Southampton, Primary Care and Population Sciences<br>Santer, Miriam; University of Southampton, Primary Care & Population Sciences<br>Lewith, George; University of Southampton<br>Moore, Michael; University of Southampton Medical School, Primary Care Medical Group<br>Little, Paul; University of Southampton, Primary Care and Population Science;

**Primary Subject Heading**:	Cardiovascular medicine
Secondary Subject Heading:	Qualitative research, General practice / Family practice
Keywords:	atrial fibrillation, QUALITATIVE RESEARCH, screening, general practice

SCHOLARONE™
Manuscripts

BMJ

I, the Submitting Author has the right to grant and does grant on behalf of all authors of the Work (as defined in the below author licence), an exclusive licence and/or a non-exclusive licence for contributions from authors who are: i) UK Crown employees; ii) where BMJ has agreed a CC-BY licence shall apply, and/or iii) in accordance with the terms applicable for US Federal Government officers or employees acting as part of their official duties; on a worldwide, perpetual, irrevocable, royalty-free basis to BMJ Publishing Group Ltd ("BMJ") its licensees and where the relevant Journal is co-owned by BMJ to the co-owners of the Journal, to publish the Work in this journal and any other BMJ products and to exploit all rights, as set out in our licence.

The Submitting Author accepts and understands that any supply made under these terms is made by BMJ to the Submitting Author unless you are acting as an employee on behalf of your employer or a postgraduate student of an affiliated institution which is paying any applicable article publishing charge ("APC") for Open Access articles. Where the Submitting Author wishes to make the Work available on an Open Access basis (and intends to pay the relevant APC), the terms of reuse of such Open Access shall be governed by a Creative Commons licence – details of these licences and which Creative Commons licence will apply to this Work are set out in our licence referred to above.

Other than as permitted in any relevant BMJ Author's Self Archiving Policies, I confirm this Work has not been accepted for publication elsewhere, is not being considered for publication elsewhere and does not duplicate material already published. I confirm all authors consent to publication of this Work and authorise the granting of this licence.

For peer review only

Patients' views about screening for atrial fibrillation (AF): a qualitative study in primary care

Mark Lown, Christopher R Wilcox, Stephanie Hughes, Miriam Santer, George Lewith‡, Michael Moore, Paul Little,

M Lown BEng, PhD, MBBS, MRCGP, GP and Clinical Lecturer, Department of Primary Care and Population Sciences, University of Southampton, Southampton.

CR Wilcox, BSc, MBBCh, Clinical Research Fellow, NIHR Clinical Research Facility, University Hospital Southampton NHS Foundation Trust, UK, Department of Primary Care and Population Sciences, University of Southampton, Southampton

S Hughes, BSc, MSc, Senior Research Assistant, Department of Primary Care and Population Sciences, University of Southampton, Southampton.

M Santer, MBBCHir, MRCGP, PhD, Associate Professor in Primary Care Research, Department of Primary Care and Population Sciences, University of Southampton, Southampton.

G Lewith MA, MD, FRCP, MRCGP, Professor of Primary care Research, Department of Primary Care and Population Sciences, University of Southampton, Southampton.

M Moore, BM, BS, MRCP, MRCGP Professor of Primary care Research, Department of Primary Care and Population Sciences, University of Southampton, Southampton.

P Little, MBBS, BA, MD, DLSHTM, MRCP, FRCGP, FMedSci, Professor of Primary care Research, Department of Primary Care and Population Sciences, University of Southampton, Southampton.

‡Deceased

Address for correspondence:

Mark Lown Department of Primary Care and Population Sciences, Aldermoor Health Centre, Aldermoor Close, University of Southampton, Southampton SO165ST.

Email: m.lown@soton.ac.uk

For peer review only

Abstract

Objectives

There has been increased interest in screening for Atrial Fibrillation (AF) with commissioned pilot schemes, ongoing large clinical trials and the emergence of inexpensive consumer single-lead ECG devices that can be used to detect AF. This qualitative study aimed to explore patients' views and understanding of AF and AF screening in order to determine acceptability and inform future recommendations.

Setting

A single primary care practice in Hampshire, UK.

Participants

15 participants (11 female) were interviewed from primary care who had taken part in an AF screening trial. A semi-structured interview guide was used flexibly, to enable the interviewer to explore any relevant topics raised by the participants. Interviews were recorded, transcribed verbatim, and analysed using inductive thematic analysis.

Results

Participants generally had an incomplete understanding of AF and conflated it with other heart problems or with raised blood pressure. With regards to potential drawbacks from screening, some participants considered anxiety and the cost of implementation, but none acknowledged potential harms associated with screening such as side effects of anti-coagulation treatment or the risk of further investigations. The screening was generally well accepted, and participants were generally in favour of engaging with prolonged screening.

Conclusions

Our study highlights that there may be poor understanding (of both the nature of AF and potential negatives of screening) amongst patients who have been screened for AF. Further work is required to determine if resources including decision aids can address this important knowledge gap, and improve clinical informed consent for AF screening.

Trial Registration: ISRCTN: ISRCTN 17495003.

For peer review only

Strengths and limitations of this study

- A strength of this study is that all the interviews were conducted by a single researcher, thus ensuring consistency.
- The study had good representation in terms of a typical screening population aged over 65 years although the high proportion of female participants may have affected the results.
- All of the participants had taken part in the SAFETY AF screening trial and those not wishing to be screened for atrial fibrillation may have had different views.
- Participants were recruited from a single surgery and may have had similar views, not representative of the wider population. However, they were form an area of low deprivation and as they had taken part in a trial were likely to be more health literate than the wider population who may have a poorer understanding of AF, although we did not record individual demographic data.
- The participants all had a negative screen which may have affected their attitudes.

For peer review only

Introduction

Atrial fibrillation (AF) is a common arrhythmia affecting around 10% of people aged over 65 in the UK[1] and is associated with an increased risk of stroke which is substantially reduced by anticoagulation.[2] Across England, it is estimated that 425,000 people are living with undiagnosed AF[3] and there has been much recent debate about screening for AF.[4] The European Cardiac Society currently recommends opportunistic screening in patients aged > 65 and consideration of systematic screening in patients aged > 75 or in those at higher stroke risk.[5] No country has yet implemented a systematic screening programme although NHS England has commissioned pilot AF screening schemes in pharmacies and in flu clinics to 'test the treatments and care models of tomorrow.[6,7]

There are ongoing, large RCTs to investigate the cost-effectiveness of AF screening and the outcomes are eagerly awaited.[8] We are also witnessing a shift in healthcare with increased consumerisation of medical devices and the emergence of relatively inexpensive consumer single-lead ECG devices and watches that can be used for AF detection.[9,10] Hence, there now exists a great need to ensure patients are well informed before undertaking any form of AF screening and to understand patient attitudes towards screening for AF. In this article, we report on an interview study that aimed to explore patient views on screening for AF.

Methods

The current qualitative study was nested within the Screening for Atrial Fibrillation using Economical and Accurate TechnologY (SAFETY) study.[11] 418 participants were recruited to the SAFETY trial from three primary care practices in the Wessex area. Individuals aged over 65 years both with and without a coded diagnosis of AF in their medical records were invited by their GP to a single nurse-led screening visit in order to test the accuracy of several devices (a blood pressure meter, a single-lead device and two ECG sensing consumer devices) for the detection of AF. Research nurses explained how to use the devices and participants were able to ask questions about AF and the devices during the visit.

Data collection

For this qualitative study, a convenience sample of 34 of the 418 trial participants not known to have AF from a single GP surgery were invited to take part in an interview.

For peer review only

Participants who consented to the qualitative study were approached sequentially (in order of randomisation to the main study) by telephone (from the first recruiting site of 3 which all had low levels of deprivation). Of these, 15 agreed to participate. We only invited participants who did not have AF as they would likely be more representative of a typical screening population. We did not record reasons for declining to participate. All the participants had previously been sent information leaflets as part of the trial invitation with detailed information on AF, screening for AF, and treatment options if AF were detected. Interviews were conducted by SH (a senior researcher with considerable qualitative research experience) via telephone, audio-recorded and transcribed verbatim, assigning ID numbers to preserve anonymity. A semi-structured interview guide was used flexibly, to enable the interviewer to explore any relevant topics raised by the participants (Table 1). The interview guide covered topics such as the patient's understanding of AF, views about AF screening (including benefits and drawbacks), opinions about the devices trialled (e.g. device comfort) and opinions about future use of the devices. The interviews were carried out between May 2017 and July 2017 and lasted around 15 minutes.

Data analysis

Interview transcripts were analysed using inductive thematic analysis (as we had little prior data or predetermined theory on AF.)[12] Transcripts were read and re-read (ML, SH, CW, MS) to identify codes, which were then organised iteratively into a coding manual by ML and CW. Main themes and sub-themes were generated independently by ML and CW then reviewed and refined through further discussions within the team, which included a mix of clinicians and academics with varying degrees of experience. The study team held regular meetings during the data collection phase and assessed the data for saturation of main themes and searched for disconfirming cases (the authors reviewed themes emerging during coding and were confident saturation had been reached when no new themes of interest were arising approximately half way through the interviews.) The sample size was appropriate and sufficient to achieve saturation of main themes[13].

For peer review only

Patient and public involvement

Patient and public representatives were involved in the design of the study from the funding application stage and in protocol development. All the study materials were developed with lay input.

Results

Participant characteristics

Of the 15 participants who took part in the qualitative study, four were male (26.7%) and the average age was 68 years (range = 65-73) (SD = 2.74). The average age of the participants in the SAFETY trial was 73.9 years and 43% were female. The index of multiple deprivation score for the participating GP practice was 17.7 (2nd quintile in England (from low levels of deprivation to high)) and the income deprivation score affecting older people (> 60 years) index was 17.3 (middle quintile). All participants were fluent in English. A number of themes emerged relating to their [1] understanding of AF, [2] attitudes to screening (in general and for AF specifically), [3] attitudes to the screening devices tested during the SAFETY study, and [4] their attitudes to undergoing prolonged screening.

Understanding of atrial fibrillation

Participants were asked to describe their understanding of AF. In interpreting responses to this question, it needs to be highlighted that all participants had received an information sheet, which described AF as 'an irregular heart rhythm that can lead to blood clots forming within the heart which can come loose and cause a stroke.' Despite this, there was considerable confusion about the nature of the condition. Although the majority of participants said they were aware that AF related to a problem with the heart, many (6/15) seemed unaware that it related to heart rhythm irregularity, and few acknowledged its association with risk of stroke or developing clots (2/15).

> *"Something wrong with your heart system…of the atrium and connecting pipes"* [P15]

> *"Well, to identify a possible stroke and high blood pressure and possible stroke and heart attack."* [P5]

For peer review only

“it’s a condition that is related to the blood circulation” [P43]

“Well if the heart isn’t functioning properly and this is a condition that could be picked up…if you have heart problems” [P65]

Attitudes to screening

When asked for their views regarding screening for atrial fibrillation, many of the participants stated positive opinions about health screening in general, with regards to early detection and saving money for the health service.

“I believe in screening as much as possible…anything that helps with picking up conditions, I think, is a good thing” [P72]

“Having more treatments or monitoring available…can’t be a bad thing” [P122]

“Saves the health service a hell of a lot of money…any kind of health screening on the NHS system is a very good idea” [P131]

Many participants raised positive opinions regarding early detection and treatment of AF specifically, and two mentioned prevention of stroke.

“I think it’s sensible to know if you’ve got AF, because then it’s possible to have some treatment” [P39]

In response to the question “What are your thoughts about screening for atrial fibrillation”: *“Well I actually think it’s a good idea; there’s a lot of heart conditions in my own family on my father’s side, and so it’s no harm to be monitored every so often, along the way”* [P53]

“I think it’s an excellent idea; better to catch a condition early if you possibly can, especially if people…[are] not aware they have it…if there’s medication or other things to stop it…I know there are so many other calls on the Health Service, but I know stroke and so on being so debilitating; if you can avoid any at all, that would be a good thing” [P125]

Most participants didn’t describe any potential downsides to screening for AF. Possible negatives that were raised by participants included anxiety and cost for the health service.

For peer review only

"It might make you anxious, but my anxiety on that score would be counteracted by - at least I know and it's now in hand, as opposed to not knowing and then it causing a complication" [P39]

"Perhaps…it might trigger people to be too anxious about their health, perhaps if they were …that sort of person" [P125]

"I suppose the cost for the NHS but, in the long-term, if you can pick something up early and correct it, it's going to be cheaper than if it's left and then down the road they are going to need more care and intervention" [P72]

"I suspect it's probably desirable…the cost or the complexity might outweigh the benefits; I'm not completely sure on that" [P122]

Participants may have misunderstood the scope of the screening test. One participant felt reassured they had a healthy heart.

"Well I suppose it was a chance to see if my heart was healthy; also it reassured me" [P65]

Attitudes to the screening devices

Some participants stated no particular preference towards any device, and felt that all were comfortable.

"Absolutely no question of them being unpleasant…they were very unintrusive and unobjectionable" [P122]

"They were all comfortable. I could have coped with any of them…if I'd been selected to use a particular type it wouldn't have bothered me" [P72]

Others participants had mixed opinions regarding the user-friendliness of the devices, and stated a preference towards those which were the least uncomfortable, least trouble, and least time-consuming to use (although their opinion as to which device was preferred differed between participants).

"I think probably the one on the finger was most convenient to use. There was another one where you had to put patches on yourself...[which] I would probably have to force myself to do at home…it would just be one of those irritating things to

For peer review only

do. There was another one…that every time you used it, you were going to have to turn on the computer, log into a site and it would upload the data onto the site... at a specific and regular time…[which would be] a bit of a faff." [P45]

"They were all fine. I think the most difficult one…where you have all the things put on you…that's the one that takes the time. But all the others seemed very, you know, quick…I think the first one was the simplest, the one with the thumbs" [P76]

"The worst one was the blood pressure cuff which was quite uncomfortable. The one…you wore it round your [rib cage] – you weren't aware you were wearing it and I think that was the best one…The handheld device was quite comfortable but…you were holding it, so whereas wearing the thing around your chest, your hands were free to do other things…and the ECG, obviously, you had to be fully engaged with that and you couldn't do other things" [P125]

Attitudes towards undertaking prolonged screening

Many participants stated that they would be happy to undergo prolonged screening using these (or similar) screening devices if it were recommended by their doctor.

"I just tend to follow advice with that sort of stuff…if it's because there was a need…it would be in my best interest then I would do yes" [P15]

"Even if it was uncomfortable…you would just do what you had to do really…I wouldn't have any hesitation if it was a health matter" [P125]

Others were more reserved about the idea of prolonged screening over a number of weeks or had specific concerns, for instance about the time required and the potential of screening to provoke anxiety or 'take over your life'.

"Well I'm not sure. It depends how many weeks we're talking about…I mean I don't have any other particular issue other than the timing" [P131]

"I'm probably a bit of an anxious person…so I might not be quite at ease…I'm not the sort of person that happily does something and just forgets…it tends to take over your life a bit…so if [it was] for two weeks or so, I might be a bit hesitant" [P130]

For peer review only

Some participants seemed unclear or doubtful about prolonged screening, i.e. they would be happy to test for AF if there was a definitive need identified by their doctor but not just if it was a matter of their age. This suggests that the test, even if they felt the test was tolerable, they may have reservations about doing this in the absence of symptoms.

> *"I mean if there was a valid reason behind it...if it was just oh you're getting to a certain age and we ought to look at it, I would probably…have a bit of a half-hearted effort at it…but if the doctor…[said that there] might be a problem here and it needs further investigation, then obviously I would take it quite seriously"* [P45]

Discussion

Principal findings

Participants expressed positive opinions towards screening for AF at an early stage and the potential to save costs although some participants mentioned that screening might provoke anxiety. The participants seemed to have an incomplete understanding of AF (despite receiving printed information sheets about AF, paroxysmal AF and anticoagulation treatment) and conflated it with other heart problems or with raised blood pressure. Although a sphygmomanometer device was employed as one of the screening devices, the other devices used in the trial measured ECG signals and participants were informed that the 12-Lead ECG they had was a definitive diagnostic test. However, more than half of those invited for the screening trial were willing to take part in the SAFETY trial[14] with the information we provided.

Importantly, when asked for their opinions on any potential drawbacks, participants did not mention the accuracy of the diagnostic test or the potential for over-diagnosis. The screening devices were generally well accepted by the participants and found to be unobtrusive, but some participants expressed concerns about comfort, user-friendliness and time taken to use the device.

Strengths and weaknesses of the study

For peer review only

A strength of this study is that participants were representative of a typical AF screening population aged over 65 years and had received information about AF and participated in screening. Patients who chose not to participate in the screening trial may however have had different views with respect to the usability and acceptance of the devices or may have chosen not to participate due to concerns over anticoagulation treatment or anxiety regarding the screening process.

A further limitation is that participants were recruited from a single surgery and may therefore hold similar views. However, as they had taken part in a trial of AF screening, were likely to have a better understanding of AF than the general population, making our finding of confusion around the topic particularly interesting.

The high proportion of female participants is a common occurrence in qualitative studies and may have affected the findings so is also a limitation. Furthermore, we were unable to explore the views of screen positive patients who may have felt they could have been better informed prior to screening and may have had different opinions on screening in general.

Findings in relation to other studies

Previous studies have identified that many patients with AF were not aware of the name of the condition[15] or that it led to an increased risk of stroke.[16] Other work has found that patients were uncertain what AF was before and after out-patient cardiology clinic appointments.[17] They also had difficulty understanding why they were treated with anti-coagulation, and why treatment was recommended lifelong.[17] Older patients, in particular, may have a poor understanding of AF[18]. This may be a key barrier to accepting anti-coagulation treatment and to future treatment adherence, which is imperative for stroke risk reduction.[19] Other work has found many patients were unaware that AF could be asymptomatic and therefore they may not be aware of paroxysmal episodes which could remain undiagnosed.[20]

Other data suggests that screening can induce anxiety in other screening programmes including breast and cervical screening[21-22] and the potential for psychological harm from being labelled with an unexpected diagnosis.[23] However, when directly asked about potential downsides of screening in this study, participants did not mention the potential risk of harm from anti-coagulation treatment, or the risk of further investigations when

For peer review only - http://bmjopen.bmj.com/site/about/guidelines.xhtml

deciding to participate, suggesting that this was not a major concern for them. This is consistent with other studies showing that many patients are unable to identify potential harms from other screening tests, and of those that did, they were mostly related to the test itself, not to further testing or treatment.[24] In contrast, patients could name benefits and tended to overestimate them.[23]

Patients do worry about potential false positive results for other screening tests such as lung cancer tests.[25] There is also concern that patients may not understand the concept of over-diagnosis[20]. However, evidence suggests that older patients may be suspicious or resistant to the concept of over-detection of other conditions.[20,26] Interestingly, there is also evidence that older adults perceived overuse to have occurred when interventions were used in the absence of symptoms (excluding cancer screening), did not improve symptoms, or against their preferences.[27]

AF screening devices have been found to be well-accepted by participants in previous large-scale trials.[28,29] However, many participants in our study stated they would be happy to undergo prolonged screening only if recommended by their doctor and some participants had specific concerns with respect to the time taken.

Practice and policy implications

There is a need to provide clear and concise information about AF, and to check patient understanding, before proceeding with screening. Checklists could be used to ensure key points have been discussed and considered, and patients may require time to weigh up the risks and benefits before deciding to proceed with screening. Decision aids have been implemented for AF treatment but have usually been designed to support clinician decisions and do not explicitly engage patients.[30] Decision aids could potentially improve patient knowledge prior to screening and have been used to improve knowledge for other screening programs.[31-34] They could be used to explain the diagnostic test, conditions that could be diagnosed, quantitative information relating to diagnostic accuracy and the risks and benefits of treatment. They could also be used to promote clarification of patients' preferences about the screening and potential consequences in order to improve patient knowledge. Health care professionals could actively ask about any potential anxiety participants may have prior to screening and be prepared to discuss these.

For peer review only

Although participants in this study did not raise concerns regarding a lack of clear information, the importance of providing information of risks as well as benefits of screening is well-established in other screening programmes.[35] For patients considering screening for AF, healthcare professionals could consider providing information about the risk of bleeding with anticoagulation treatment if AF were detected, in addition to potential benefits. A discussion of available treatment options and lifestyle modifications before undergoing testing might also ensure the potential for future treatment adherence if AF was diagnosed. Patients could also be informed about the specificity of the screening test, and reminded that screening will not provide information on general heart function or cardiovascular risk in order to avoid false reassurance.

Future research

Further research should focus on screen positive patients to determine their views on information provision prior to screening and their wider opinions on screening for AF. Future research should also include a wider demographic of patients. There is also a need to understand how pre-screening information and discussions influence patients' decisions to undergo screening for AF.

Conclusions

Our study highlights that, even amongst patients who have been screened for AF, there may be poor understanding of both the nature of AF and potential negatives of screening. Further work is required to determine if resources including decision aids can address this important knowledge gap, and help patients make informed decisions around AF screening.

Word count 3296

Acknowledgements: The authors would like to thank all the participants who took part in this study: without them this study would not have been possible.

Contributions: ML, GL, MM, PL designed the study. SH conducted the interviews. ML & CW coded and analysed the interview data. ML, CW, SH & MS contributed to the interpretation

For peer review only

of the data. ML drafted the manuscript and all authors contributed to the review and editing of the manuscript.

Funding: This study/project is funded by the National Institute for Health Research (NIHR) School for Primary Care Research (project reference 318). The views expressed are those of the authors and not necessarily those of the NIHR or the Department of Health and Social Care.

Competing Interests: None declared.

Ethics approval: The study complies with the declaration of Helsinki, and the protocol was approved by the London - City & East Research Ethics Committee in June 2016 (ref 16/LO/1173). Informed consent was obtained from all participants (trial registration ISRCTN: 17495003).

Data availability statement: No data are available.

References

1. Cowan C, Healicon R, Robson I, et al. The use of anticoagulants in the management of atrial fibrillation among general practices in England. *Heart* 2013; **99(16)**: 1166-72.
2. Hart RG, Pearce LA, Aguilar MI. Meta-analysis: antithrombotic therapy to prevent stroke in patients who have nonvalvular atrial fibrillation. *Ann Intern Med.* 2007; **146(12)**:857-67.
3. Reiffel JA, Atrial fibrillation and stroke: epidemiology. *Am J Med.* 2014; **127(4)**:e15-6.
4. Lown M, Moran P. Should we screen for atrial fibrillation? *BMJ.* 2019 Feb 13;364:l43.
5. Mairesse GH, Moran P, Van Gelder IC, Elsner C, Rosenqvist M, Mant J, Banerjee A, Gorenek B, Brachmann J, Varma N, Glotz de Lima G, Kalman J, Claes N, Lobban T, Lane D, Lip GYH, Boriani G; ESC Scientific Document Group. Screening for atrial fibrillation: a European Heart Rhythm Association (EHRA) consensus document endorsed by the Heart Rhythm Society (HRS), Asia Pacific Heart Rhythm Society (APHRS), and Sociedad Latinoamericana de Estimulación Cardíaca y Electrofisiología (SOLAECE). *Europace.* 2017; **19(10)**:1589-1623.
6. Care City launches life-saving atrial fibrillation pathway. https://nhsaccelerator.com/care-city-launches-life-saving-atrial-fibrillation-pathway/. Accessed 01/03/2019.
7. Atrial Fibrillation Check in Influenza Vaccination Programme. http://www.londonscn.nhs.uk/wp-content/uploads/2017/07/heywood-middleton-and-rochdale-ccg.pdf Accessed 01/03/2019.
8. Nuffield Department of Primary Care Health Sciences. Upcoming trial involving 120 000 patients to investigate screening for atrial fibrillation. https://www.phc.ox. ac.uk/news/new-research-to-investigate-whether-screening-can-prevent-one-in-ten-strokes.Accessed 01/03.2019.
9. Gropler MRF, Dalal AS, Van Hare GF, Silva JNA. Can smartphone wireless ECGs be used to accurately assess ECG intervals in pediatrics? A comparison of mobile health monitoring to standard 12-lead ECG. *PLoS One*. 2018;**13(9)**:e0204403.
10. Apple Watch Series 4: Proactive Health Monitor. https://www.apple.com/apple-watch-series-4/health/ accessed 01/04/2019.
11. Lown M, Yue A, Lewith G, Little P, Moore M. Screening for Atrial Fibrillation using Economical and accurate TechnologY (SAFETY)-a pilot study. *BMJ Open*. 2017;**7(1)**:e013535.

For peer review only

12. Braun V, Clarke V. Using Thematic Analysis in Psychology. *Qualitative Research in Psychology* 2006. **3(2)**:77-101
13. Guest G, Bruce A, Johnson L. How many interviews are enough? An experiment with data saturation and variability. *Field Methods* 2006; **18(1)**: 59-82.
14. Lown M, Yue AM, Shah BN, Corbett SJ, Lewith G, Stuart B, Garrard J, Brown M, Little P, Moore M. Screening for Atrial Fibrillation Using Economical and Accurate Technology (From the SAFETY Study). Am J Cardiol. 2018 Oct 15;122(8):1339-1344.
15. Lip GY, Kamath S, Jafri M, Mohammed A, Bareford D. Ethnic differences in patient perceptions of atrial fibrillation and anticoagulation therapy: the West Birmingham Atrial Fibrillation Project. *Stroke.* 2002; **33(1)**:238-42
16. McCabe PJ, Schad S, Hampton A, Holland DE. Knowledge and self-management behaviors of patients with recently detected atrial fibrillation. *Heart Lung.* 2008; **37(2)**:79-90.
17. Thrysoee L, Strömberg A, Brandes A, Hendriks JM. Management of newly diagnosed atrial fibrillation in an outpatient clinic setting-patient's perspectives and experiences. *J Clin Nurs.* 2018; **27(3-4)**:601-611.
18. Lindberg T, Sanmartin Berglund J, Elmståhl S, Bohman DM. Older individuals' need for knowledge and follow-up about their chronic atrial fibrillation, lifelong medical treatment and medical controls. *Scand J Caring Sci.* 2017; **31(4)**:1022-1030.
19. Salmasi S, Kwan L, MacGillivray J, Bansback N, De Vera MA, Barry AR, Harrison MJ, Andrade J, Lynd LD, Loewen P. Assessment of atrial fibrillation patients' education needs from patient and clinician perspectives: A qualitative descriptive study. *Thromb Res.* 2019;**173**:109-116.
20. Salmasi S, De Vera MA, Barry A, Bansback N, Harrison M, Lynd LD, Loewen PS. Assessment of Condition and Medication Knowledge Gaps Among Atrial Fibrillation Patients: A Systematic Review and Meta-analysis. *Ann Pharmacother*. 2019 Mar 2.
21. Mathioudakis AG, Salakari M, Pylkkanen L, Saz-Parkinson Z, Bramesfeld A, Deandrea S, Lerda D, Neamtiu L, Pardo-Hernandez H, Solà I, Alonso-Coello P. Systematic review on women's values and preferences concerning breast cancer screening and diagnostic services. *Psychooncology*. 2019 Feb 27.
22. Patel H, Moss EL, Sherman SM. HPV primary cervical screening in England: Women's awareness and attitudes. *Psychooncology*. 2018; **27(6)**:1559-1564.
23. Cotter AR, Vuong K, Mustelin L, Yang Y, Rakhmankulova M, Barclay CJ, Harris RP. Do psychological harms result from being labelled with an unexpected diagnosis of abdominal aortic aneurysm or prostate cancer through screening? A systematic review. *BMJ Open*. 2017; **7(12)**:e017565
24. Sutkowi-Hemstreet A, Vu M, Harris R, Brewer NT, Dolor RJ, Sheridan SL. Adult Patients' Perspectives on the Benefits and Harms of Overused Screening Tests: a Qualitative Study. *J Gen Intern Med.* 2015; **30(11):**1618-26.
25. Carter-Harris L, Brandzel S, Wernli KJ, Roth JA, Buist DSM. A qualitative study exploring why individuals opt out of lung cancer screening. *Fam Pract.* 2017;**34(2)**:239-244
26. Pappadis MR, Volk RJ, Krishnan S, Weller SC, Jaramillo E, Hoover DS, Giordano SH, Tan A, Sheffield KM, Housten AJ, Goodwin JS. Perceptions of overdetection of breast cancer among women 70 years of age and older in the USA: a mixed-methods analysis. *BMJ Open*. 2018**; 8(6)**:e022138.
27. Green AR, Tung M, Segal JB. Older Adults' Perceptions of the Causes and Consequences of Healthcare Overuse: A Qualitative Study. *J Gen Intern Med*. 2018;**33(6)**:892-897.
28. Lowres N, Krass I, Neubeck L, Redfern J, McLachlan AJ, Bennett AA, Freedman SB. Atrial fibrillation screening in pharmacies using an iPhone ECG: a qualitative review of implementation. *Int J Clin Pharm.* 2015;**37(6)**:1111-20.
29. Svennberg E, Engdahl J, Al-Khalili F, Friberg L, Frykman V, Rosenqvist M. Mass Screening for Untreated Atrial Fibrillation: The STROKESTOP Study. *Circulation*. 2015;**131(25)**:2176-84.
30. Probst MA, Noseworthy PA, Brito JP, Hess EP. Shared Decision-Making as the Future of Emergency Cardiology. *Can J Cardiol*. 2018;**34(2)**:117-124.
31. Perestelo-Perez L, Rivero-Santana A, Torres-Castaño A, Ramos-Garcia V, Alvarez-Perez Y, Gonzalez-Hernandez N, Buron A, Pignone M, Serrano-Aguilar P. Effectiveness of a decision aid for promoting colorectal cancer screening in Spain: a randomized trial. *BMC Med Inform Decis Mak.* 2019;**19(1)**:8.
32. Smith SK, Cai A, Wong M, Sousa MS, Peate M, Welsh A, Meiser B, Kaur R, Halliday J, Lewis S, Trevena L, Yanes T, Barlow-Stewart K, Barclay M. Improving women's knowledge about prenatal screening in the era

For peer review only

of non-invasive prenatal testing for Down syndrome - development and acceptability of a low literacy decision aid. *BMC Pregnancy Childbirth*. 2018;**18(1)**:499.
33. Wang Y, Anazodo A, Logan S. Systematic review of fertility preservation patient decision aids for cancer patients. *Psychooncology*. 2019 Mar;**28(3):**459-467.
34. Ivlev I, Jerabkova S, Mishra M, Cook LA, Eden KB. Prostate Cancer Screening Patient Decision Aids: A Systematic Review and Meta-analysis. *Am J Prev Med.* 2018;**55(6)**:896-907.
35. Stefanek ME. Uninformed compliance or informed choice? A needed shift in our approach to cancer screening. J Natl Cancer Inst. 2011 Dec 21;103(24):1821-6.

For peer review only

Interview Schedule
Can you describe your understanding of atrial fibrillation?
What are your views about screening for atrial fibrillation?<br>- What do you think the positives of screening are?<br>- What do you think the negatives of screening are?
What did you think about the study information given to you?
Did you have any reservations about taking part, and if so, can you tell me a bit about them?
Do you have any regrets about taking part? If so, can you tell me a bit about them?
Could you tell me a bit about the devices you tried as part of the SAFETY trial?
How comfortable did you find the devices?
How would you feel about wearing or using the devices for a few weeks for screening?
Is there anything else you'd like to share about your participation in the trial, or your thoughts about AF

Table 1 – Interview Schedule

For peer review only

Standards for Reporting Qualitative Research (SRQR)*

http://www.equator-network.org/reporting-guidelines/srqr/

Title and abstract

	Page/line no(s).
**Title** - Concise description of the nature and topic of the study Identifying the study as qualitative or indicating the approach (e.g., ethnography, grounded theory) or data collection methods (e.g., interview, focus group) is recommended	1
**Abstract** - Summary of key elements of the study using the abstract format of the intended publication; typically includes background, purpose, methods, results, and conclusions	2

Introduction

**Problem formulation** - Description and significance of the problem/phenomenon studied; review of relevant theory and empirical work; problem statement	4
**Purpose or research question** - Purpose of the study and specific objectives or questions	4

Methods

**Qualitative approach and research paradigm** - Qualitative approach (e.g., ethnography, grounded theory, case study, phenomenology, narrative research) and guiding theory if appropriate; identifying the research paradigm (e.g., postpositivist, constructivist/ interpretivist) is also recommended; rationale**	4-5
**Researcher characteristics and reflexivity** - Researchers' characteristics that may influence the research, including personal attributes, qualifications/experience, relationship with participants, assumptions, and/or presuppositions; potential or actual interaction between researchers' characteristics and the research questions, approach, methods, results, and/or transferability	5
**Context** - Setting/site and salient contextual factors; rationale**	5
**Sampling strategy** - How and why research participants, documents, or events were selected; criteria for deciding when no further sampling was necessary (e.g., sampling saturation); rationale**	5
**Ethical issues pertaining to human subjects** - Documentation of approval by an appropriate ethics review board and participant consent, or explanation for lack thereof; other confidentiality and data security issues	13
**Data collection methods** - Types of data collected; details of data collection procedures including (as appropriate) start and stop dates of data collection and analysis, iterative process, triangulation of sources/methods, and modification of procedures in response to evolving study findings; rationale**	5

For peer review only

For peer review only - http://bmjopen.bmj.com/site/about/guidelines.xhtml

**Data collection instruments and technologies** - Description of instruments (e.g., interview guides, questionnaires) and devices (e.g., audio recorders) used for data collection; if/how the instrument(s) changed over the course of the study	5
**Units of study** - Number and relevant characteristics of participants, documents, or events included in the study; level of participation (could be reported in results)	5
**Data processing** - Methods for processing data prior to and during analysis, including transcription, data entry, data management and security, verification of data integrity, data coding, and anonymization/de-identification of excerpts	5
**Data analysis** - Process by which inferences, themes, etc., were identified and developed, including the researchers involved in data analysis; usually references a specific paradigm or approach; rationale**	5
**Techniques to enhance trustworthiness** - Techniques to enhance trustworthiness and credibility of data analysis (e.g., member checking, audit trail, triangulation); rationale**	5

Results/findings

**Synthesis and interpretation** - Main findings (e.g., interpretations, inferences, and themes); might include development of a theory or model, or integration with prior research or theory	6-9
**Links to empirical data** - Evidence (e.g., quotes, field notes, text excerpts, photographs) to substantiate analytic findings	6-9

Discussion

**Integration with prior work, implications, transferability, and contribution(s) to the field** - Short summary of main findings; explanation of how findings and conclusions connect to, support, elaborate on, or challenge conclusions of earlier scholarship; discussion of scope of application/generalizability; identification of unique contribution(s) to scholarship in a discipline or field	10-13
**Limitations** - Trustworthiness and limitations of findings	3

Other

**Conflicts of interest** - Potential sources of influence or perceived influence on study conduct and conclusions; how these were managed	13
**Funding** - Sources of funding and other support; role of funders in data collection, interpretation, and reporting	13

*The authors created the SRQR by searching the literature to identify guidelines, reporting standards, and critical appraisal criteria for qualitative research; reviewing the reference lists of retrieved sources; and contacting experts to gain feedback. The SRQR aims to improve the transparency of all aspects of qualitative research by providing clear standards for reporting qualitative research.

For peer review only

**The rationale should briefly discuss the justification for choosing that theory, approach, method, or technique rather than other options available, the assumptions and limitations implicit in those choices, and how those choices influence study conclusions and transferability. As appropriate, the rationale for several items might be discussed together.

Reference:

O'Brien BC, Harris IB, Beckman TJ, Reed DA, Cook DA. **Standards for reporting qualitative research: a synthesis of recommendations.** *Academic Medicine*, Vol. 89, No. 9 / Sept 2014 DOI: 10.1097/ACM.0000000000000388

For peer review only

For peer review only - http://bmjopen.bmj.com/site/about/guidelines.xhtml
